# Synthesis of heteroglycoclusters by using orthogonal chemoselective ligations

**DOI:** 10.3762/bjoc.8.47

**Published:** 2012-03-20

**Authors:** Baptiste Thomas, Michele Fiore, Isabelle Bossu, Pascal Dumy, Olivier Renaudet

**Affiliations:** 1 Département de Chimie Moléculaire, UMR-CNRS 5250 & ICMG FR 2607, Université Joseph Fourier, PB 53, 38041 Grenoble Cedex 9, France; 2 Institut Universitaire de France, 103 Boulevard Saint-Michel, 75005 Paris, France

**Keywords:** chemoselective ligation, click chemistry, cyclopeptide, heteroglycocluster, oxime

## Abstract

Synthetic heteroglycoclusters are being subjected to increasing interest due to their potential to serve as selective ligands for carbohydrate-binding proteins. In this paper, we describe an expedient strategy to prepare cyclopeptides displaying well-defined distributions and combinations of carbohydrates. By using both oxime ligation and copper(I)-catalyzed alkyne–azide cycloaddition, two series of compounds bearing binary combinations of αMan, αFuc or βLac in an overall tetravalent presentation, and either 2:2 or 3:1 relative proportions, have been prepared.

## Introduction

Multivalent interactions between carbohydrates and proteins play key roles in diverse biological events, including fertilization, cell–cell communication, host–pathogen interactions, immune response and cancer metastasis [1]. Synthetic molecules displaying multiple copies of a sugar binding motif, called (homo)glycoclusters, represent attractive tools for studying these complex recognition processes as well as for developing biological applications, for example, the inhibition of infections by pathogens such as viruses or bacteria [2–5]. In a suitable density and spatial display, clusters of carbohydrate indeed allow multiple contacts with a target protein, thus increasing avidity by means of the “glycoside cluster effect” [6–7]. While the recent progress in glycomics has led to the design of glycoclusters active at nanomolar concentration [8–10], the achievement of selective binding remains challenging because of the close structural similarities of the binding sites of proteins specific for the same carbohydrate moiety.

**Figure 1:**
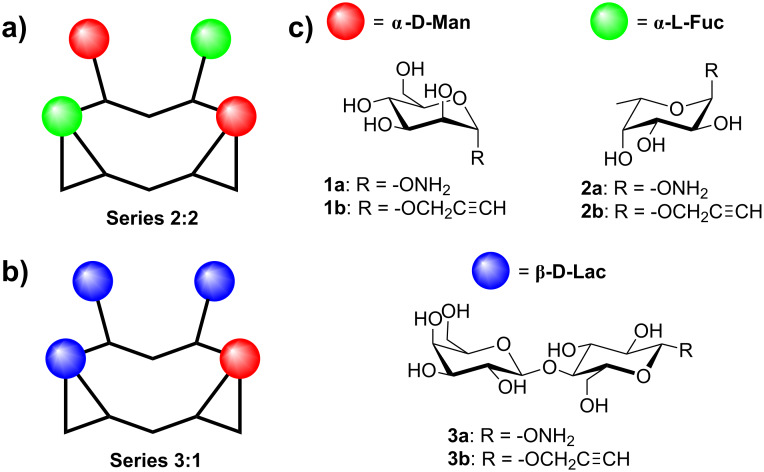
(a) Schematic representation of a heteroglycocluster of the 2:2 series containing Man and Fuc. (b) Schematic representation of a heteroglycocluster of the 3:1 series containing Lac and Man. (c) Structure of carbohydrates used for the construction of heteroglycoclusters.

Interestingly, recent reports have highlighted that the association of different sugar units instead of a single motif, namely heteroglycocluster, reflects the presence of sugars found in native biological systems more closely than homoglycocluster does. Although recognition mechanisms are not fully understood, these studies suggest that heteroglycoclusters should interact with proteins through distinct binding sites, which may influence both affinity and selectivity [11–25]. In this context, we previously reported a combinatorial procedure to prepare libraries of heteroglycoclusters displaying sugars and/or amino acids at randomized positions on a topological cyclopeptide scaffold [26]. Deconvolution of the resulting libraries by affinity chromatography allowed the selection of heteroglycoclusters that were proved to be useful for exploring the surrounding regions of the binding pocket in a model lectin. Although it is easy to handle, this combinatorial procedure leads to the formation of inseparable mixtures of regioisomers, which precludes their utilization for further assays with relevant biological targets. In order to circumvent this drawback, we herein report the synthesis of similar heteroglycoclusters by using a protocol based on orthogonal chemoselective ligations. Two series of compounds containing different combinations of two different sugars have been designed (Figure 1). In one series (heteroglycocluster 2:2), two αMan, αFuc and/or βLac are conjugated at alternate positions into the tetravalent cyclopeptide sequence, whereas the second series (heteroglycocluster 3:1) contains one of a given sugar and three of another.

## Results and Discussion

**Scheme 1:**
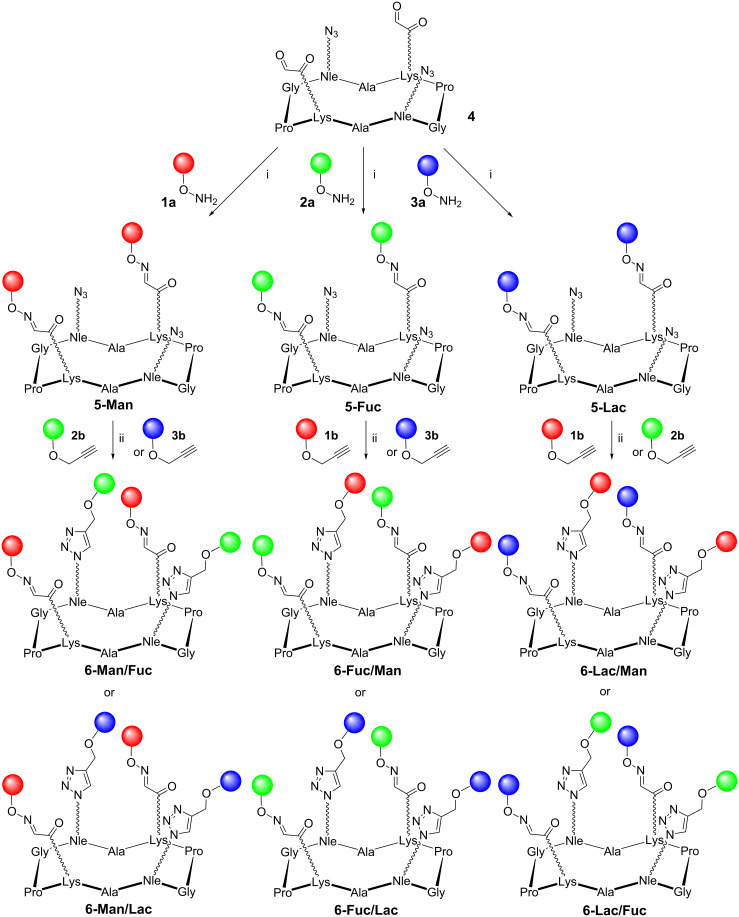
Synthesis of heteroglycoclusters of the 2:2 series. Reagents and conditions: (i) 0.1% TFA in H_2_O; (ii) Cu micropowder, *t*-BuOH, AcONH_4_ 100 mM pH 7.4 (1:1, *v*/*v*). The wavy bond represents the aliphatic part (i.e., (CH_2_)_4_) of the lysine (Lys) and the norleucine (Nle) side chain.

Glycoclusters are classically constructed from a molecular scaffold containing multiple anchoring sites that can be functionalized with sugars by using a single coupling reaction. By contrast, the chemical access of heteroglycoclusters is not trivial because it requires the controlled conjugation of different sugars at a precise position into the scaffold to obtain a well-defined distribution. Taking advantage of our experience in bioconjugation methods, we decided to explore two chemoselective strategies to achieve this purpose. We first selected the oxime ligation since we have previously used this approach successfully for the preparation of sophisticated molecular systems, such as synthetic vaccines [27–28], immunomodulators [29], lectin ligands [30] or vectors of hepatocytes [31]. As the second strategy, we have chosen the well-known copper(I)-catalyzed alkyne–azide cycloaddition (CuAAC) [32–33], which is intensively exploited for the conjugation of sugars to both molecular and biological systems [34–35]. Besides being fully compatible with carbohydrate and peptide chemistries, oximation and CuAAC reactions offer the advantage of being orthogonal [36–37], therefore allowing a controlled assembly process with a minimized risk of side reactions. The 2:2 series of heteroglycoclusters was prepared from the aminooxy [38–40] and propargyl [41] glycosides **1**-**3** (Figure 1) and the cyclopeptide **4** (Scheme 1). This scaffold containing two lysines (Lys) functionalized with an aldehyde and two norleucines (Nle) bearing an azide group was prepared by using a strategy adapted from the procedure described previously [42].

In the first step, two copies of aminooxy αMan **1a**, αFuc **2a** and βLac **3a** were coupled to **4**, affording divalent oxime-linked compounds **5-Man**, **5-Fuc**, and **5-Lac**, respectively. The oxime ligation was performed at 37 °C in aqueous acidic buffer with 2 equiv of sugars per anchoring site. After 3 h, complete reactions were observed by analytical HPLC. The excess of sugar was then quenched by the addition of acetone, and the resulting crude mixtures were used for CuAAC without further purification. The efficiency of CuAAC clearly depends on the experimental conditions [34–35]. The choice of the solvent and of the copper(I) catalyst (delivered either using CuI, copper micropowder, or CuSO_4_ and sodium ascorbate as reducing agent) and the utilization of microwave or ultrasonic irradiation are parameters that can influence the reaction kinetics, improve the yields and sometimes prevent side reactions. In a previous study, we observed that a tetravalent glycocluster can be obtained in good yields and as a unique 1,4-regioisomer by using a catalytic amount of copper micropowder in a mixture of isopropanol and ammonium acetate buffer [42]. Therefore we decided to follow similar conditions in this study with propargyl glycosides αMan **1b**, αFuc **2b** and βLac **3b**. Here again, each reaction occurred quantitatively, as shown in RP-HPLC profiles of the crude reaction mixtures (see Supporting Information File 1). After removal of solid copper by filtration and semipreparative HPLC, six tetravalent heteroglycoclusters combining two sugars (e.g., **6-Man/Fuc**, **6-Man/Lac**, **6-Fuc/Man**, **6-Fuc/Lac**, **6-Lac/Man** and **6-Lac/Fuc**) were obtained in excellent conversion rate and purity and unambiguously characterized by mass spectrometry (Table 1).

**Table 1:** Outcome of the orthogonal ligation procedure.

compound	yield^a^	MS calcd^b^	MS found^c^	*t*_R_ (min)^d^

**6-Man/Fuc**	83%	1885.9 (C_78_H_124_N_20_O_34_)	1886.0	7.79
**6-Man/Lac**	99%	2242.9 (C_90_H_145_N_20_O_46_)	2242.3	7.43
**6-Fuc/Man**	98%	1885.9 (C_78_H_124_N_20_O_34_)	1886.0	7.73
**6-Fuc/Lac**	98%	2210.0 (C_90_H_145_N_20_O_44_)	2210.3	7.62
**6-Lac/Man**	94%	2242.9 (C_90_H_145_N_20_O_46_)	2242.2	7.31
**6-Lac/Fuc**	93%	2210.0 (C_90_H_145_N_20_O_44_)	2210.3	7.60
**8-Man/Fuc**	85%	1880.9 (C_79_H_126_N_21_O_32_)	1881.1	7.88
**8-Man/Lac**	87%	2415.0 (C_97_H_156_N_21_O_50_)	2415.4	7.30
**8-Fuc/Man**	91%	1912.9 (C_79_H_126_N_21_O_34_)	1913.2	7.66
**8-Fuc/Lac**	90%	2400.0 (C_97_H_156_N_21_O_49_)	2399.3	7.46
**8-Lac/Man**	89%	2092.0 (C_85_H_136_N_21_O_40_)	2091.2	7.46
**8-Lac/Fuc**	91%	2043.9 (C_85_H_136_N_21_O_37_)	2043.1	7.83

^a^Yields were calculated by integrating the peak corresponding to the expected compound in the crude HPLC profile. Isolated yields are given in the Experimental section. ^b^Calculated mass for [M + H]^+^. ^c^MS analysis was performed by electrospray ionization method in positive mode. ^d^RP-HPLC retention time using a linear gradient A/B, 95:5 to 0:100 in 20 min, flow: 1.0 mL/ min, λ = 214 nm and 250 nm (column: nucleosil 300-5 C_18_; solvent A: 0.09% TFA in H_2_O, solvent B: 0.09% TFA in 90% acetonitrile).

**Scheme 2:**
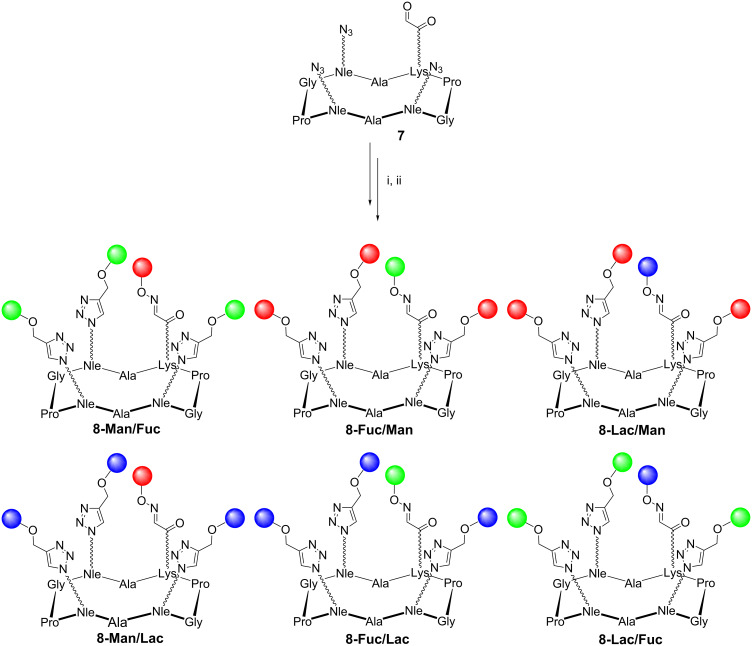
Synthesis of heteroglycoclusters of the 3:1 series. Reagents and conditions: (i) **1a**, **2a** or **3a**, 0.1% TFA in H_2_O; (ii) **1b**, **2b** or **3b**, Cu micropowder, *t*-BuOH, AcONH_4_ 100 mM pH 7.4 (1:1, *v*/*v*).

To demonstrate the reliability of our protocol, a new series of 3:1 heteroglycoclusters was prepared from the same carbohydrate building blocks and the cyclopeptide **7**. Similar experimental conditions were followed, with the exception of the stoichiometry of reagents (see Experimental section). In this series, one oxime linkage was formed from **7** by using aminooxy αMan **1a**, αFuc **2a** and βLac **3a**, and three CuAAC reactions were subsequently performed with propargyl glycosides αMan **1b**, αFuc **2b** and βLac **3b** as described above (Scheme 2).

No difference in reactivity from the previous 2:2 series was observed, thereby confirming the efficiency of this strategy for the preparation of well-defined heteroglycoclusters.

## Conclusion

In this paper we have described an expedient and controlled assembly protocol to prepare heteroglycoclusters similar to those obtained previously from randomized combinatorial libraries [26]. Following two orthogonal chemoselective reactions, cyclopeptides **4** and **7** were successively reacted with aminooxy glycosyls **1**–**3a** and propargyl glycosides **1**–**3b.** Twelve novel oxime and triazole linked-heteroglycoclusters displaying well-defined distributions and combinations of carbohydrates were thus obtained in excellent yields and purity.

## Experimental

### Standard procedures for the heteroglycocluster assembly

#### Series 2:2

A solution of **4** (4.0 mg, 3.4 µmol) and **2a** (2.6 mg, 14.2 µmol, 4 equiv) was stirred at 37 °C in 0.1% TFA in H_2_O (400 µL). After 3 h, analytical HPLC revealed the total conversion of **4a** into **5-Fuc**. Analytical HPLC: *t*_R_ 9.34 min (gradient: 5 to 100% B in 20 min); ESI^+^–MS (*m*/*z*): [M + Na]^+^ calcd for C_60_H_96_N_20_O_22_Na, 1471.7; found, 1471.8. After the addition of acetone (100 µL) to the crude, the reaction mixture was lyophilized, then resuspended with *t*-BuOH/AcONH_4_ 100 mM pH 7.4 (500 µL, 1:1 *v*/*v*). Compound **1b** (4.0 mg, 10.5 µmol, 3 equiv) and copper micropowder (455 µg, 7.0 µmol) were next added to the solution and the resulting mixture was left under stirring at room temperature. After 4 h, copper was removed by centrifugation and the supernatant purified by semipreparative HPLC. Compound **6-Fuc/Man** was obtained in 91% yield (5.9 mg). Analytical RP-HPLC: *t*_R_ 7.73 min (gradient: 5 to 100% B in 20 min); ^1^H NMR (400 MHz, D_2_O) δ 8.07 (s, 1H, H_trz_), 8.06 (s, 1H, H_trz_), 7.81 (s, 1H, H_ox_), 7.80 (s, 1H, H_ox_), 5.62 (d, *J*_1,2_ = 4.0 Hz, 1H, H-1_Fuc_), 5.60 (d, *J*_1,2_ = 4.0 Hz, 1H, H-1_Fuc_), 4.99 (bs, 2H, 2H-1_Man_), 4.86–4.68 (m, 6H, 2H_αLys/Nle_, 2CH_2propargyl_), 4.47–4.35 (m, 10H, 2H_αLys/Nle_, 2CH_2εNle_, 2H_αAla_, 2H_αPro_), 4.16–3.63 (m, 28H, 2CH_2αGly_, 2CH_2δPro_, 2H-2_Fuc_, 2H-3_Fuc_, 2H-4_Fuc_, 2H-5_Fuc_, 2H-2_Man_, 2H-3_Man_, 2H-4_Man_, 2H-5_Man_, 2CH_2_-6_Man_), 3.36–3.20 (m, 4H, 4H_εLys_), 2.39–2.29 (m, 2H, 2H_βPro_), 2.16–1.27 (m, 36H, 4CH_2βLys/Nle_, 4CH_2δLys/Nle_, 4CH_2γLys/Nle_, 2H_βPro_, 2CH_2γPro_, 2CH_3Ala_), 1.22 (d, *J*_5,6_ = 6.0 Hz, 6H, 2CH_3Fuc_); ESI^+^–MS (*m*/*z*): [M + H]^+^ calcd for C_78_H_124_N_20_O_34_, 1885.9; found, 1886.0. Compounds **6-Man/Fuc**, **6-Man/Lac**, **6-Fuc/Lac**, **6-Lac/Man** and **6-Lac/Fuc** were prepared following the same experimental conditions.

#### Series 3:1

A solution of **7** (6.6 mg, 5.9 µmol) and **2a** (2.2 mg, 1.2 µmol, 2 equiv) was stirred at 37 °C in 0.1% TFA in H_2_O (600 µL). After 3 h, analytical HPLC revealed the total conversion of **7** into the corresponding monovalent intermediate. Analytical HPLC *t*_R_ 10.57 min (gradient: 5 to 100% B in 20 min); ESI^+^–MS (*m*/*z*): [M + H]^+^ calcd for C_52_H_84_N_21_O_17_, 1274.6; found, 1274.8. After the addition of acetone (100 µL) to the crude, the reaction mixture was lyophilized, then resuspended with *t*-BuOH/AcNH_4_ 100 mM pH 7.4 (600 µL, 1:1 *v*/*v*). Compound **1b** (10 mg, 27 µmol, 4.5 equiv) and copper micropowder (600 µg, 10 µmol) were next added to the solution and the resulting mixture was left under stirring at room temperature. After 4 h, copper was removed by centrifugation and the supernatant purified by semipreparative HPLC. Compound **8-Fuc/Man** was obtained in 88% yield (10.0 mg). Analytical RP-HPLC: *t*_R_ 7.66 min (gradient: 5 to 100% B in 20 min); ^1^H NMR (400 MHz, D_2_O) δ 8.05 (s, 1H, H_trz_), 8.02 (s, 2H, H_trz_), 7.79 (s, 1H, H_ox_), 5.61 (d, *J*_1,2_ = 4.0 Hz, 1H, H-1_Fuc_), 5.60 (d, *J*_1,2_ = 4.0 Hz, 1H, H-1_Fuc_), 4.99 (bs, 3H, 3H-1_Man_), 4.85–4.66 (m, 8H, 2H_αLys/Nle_, 3CH_2propargyl_), 4.48–4.32 (m, 12H, 2H_αLys/Nle_, 3CH_2εNle_, 2H_αAla_, 2H_αPro_), 4.15–3.64 (m, 30H, 2CH_2αGly_, 2CH_2δPro_, H-2_Fuc_, H-3_Fuc_, H-4_Fuc_, H-5_Fuc_, 3H-2_Man_, 3H-3_Man_, 3H-4_Man_, 3H-5_Man_, 3CH_2_-6_Man_), 3.29–3.19 (m, 2H, 2H_εLys_), 2.40–2.30 (m, 2H, 2H_βPro_), 2.15–1.28 (m, 36H, 4CH_2βLys/Nle_, 4CH_2δLys/Nle_, 4CH_2γLys/Nle_, 2H_βPro_, 2CH_2γPro_, 2CH_3Ala_), 1.22 (d, *J*_5,6_ = 6.6 Hz, 3H, CH_3Fuc_); ESI^+^–MS (*m*/*z*): [M + H]^+^ calcd for C_79_H_126_N_21_O_34_, 1912.9; found, 1913.2. Compounds **8-Man/Fuc**, **8-Man/Lac**, **8-Fuc/Lac**, **8-Lac/Man** and **8-Lac/Fuc** were prepared under the same experimental conditions.

## Supporting Information

The Supporting Information file contains analytical details of all heteroglycoclusters of series 2:2 (**6-Man/Fuc**, **6-Man/Lac**, **6-Fuc/Man**, **6-Fuc/Lac**, **6-Lac/Man** and **6-Lac/Fuc**) and 3:1 (**8-Man/Fuc**, **8-Man/Lac**, **8-Fuc/Man**, **8-Fuc/Lac**, **8-Lac/Man** and **8-Lac/Fuc**) described in this article.

File 1 Crude RP-HPLC profiles and ESI-MS spectra for the heteroglycoclusters.
