# Supplementary material for: Synthesis of heteroglycoclusters by using orthogonal chemoselective ligations
Source: Beilstein J Org Chem. 2012 Mar 20;8:421–7. doi: 10.3762/bjoc.8.47 (PMC3326620; doi:10.3762/bjoc.8.47)

Supporting Information

for

Synthesis of heteroglycoclusters by using orthogonal chemoselective ligations

Baptiste Thomas[1], Michele Fiore[1], Isabelle Bossu[1], Pascal Dumy[1] and Olivier Renaudet*[1,2]

Address: [1]Département de Chimie Moléculaire, UMR-CNRS 5250 & ICMG FR 2607, Université Joseph Fourier, PB 53, 38041 Grenoble Cedex 9, France and [2]Institut Universitaire de France, 103 boulevard Saint-Michel, 75005 Paris, France.

Email: Olivier Renaudet* - olivier.renaudet@ujf-grenoble.fr

*Corresponding author

Crude RP-HPLC profiles and ESI-MS spectra for the heteroglycoclusters

Table of contents:

1. Analytical data for compound **5-Man** S3
2. Analytical data for compound **5-Fuc** S4
3. Analytical data for compound **5-Lac** S5
4. Analytical data for compound **6-Man/Fuc** S6
5. Analytical data for compound **6-Man/Lac** S7
6. Analytical data for compound **6-Fuc/Man** S8–S9
7. Analytical data for compound **6-Fuc/Lac** S10
8. Analytical data for compound **6-Lac/Man** S11

9.	Analytical data for compound **6-Lac/Fuc**	S12
10.	Analytical data for compound **7-Man**	S13
11.	Analytical data for compound **7-Fuc**	S14
12.	Analytical data for compound **7-Lac**	S15
13.	Analytical data for compound **8-Man/Fuc**	S16
14.	Analytical data for compound **8-Man/Lac**	S17
15.	Analytical data for compound **8-Fuc/Man**	S18–S19
16.	Analytical data for compound **8-Fuc/Lac**	S20
17.	Analytical data for compound **8-Lac/Man**	S21
18.	Analytical data for compound **8-Lac/Fuc**	S22

General details

All chemical reagents were purchased from Aldrich (Saint Quentin Fallavier, France) or Acros (Noisy-Le-Grand, France) and were used without further purification. Protected amino acids and Fmoc-Gly-Sasrin resin were obtained from Advanced ChemTech Europe (Brussels, Belgium), Bachem Biochimie SARL (Voisins-Les-Bretonneux, France) and France Biochem S.A. (Meudon, France). PyBOP was purchased from France Biochem. Reaction progress was monitored by reverse-phase HPLC on Waters equipment by using C_{18} columns. Analytical and preparative separation was carried out at 1.0 mL/min (EC 125/3 nucleosil 300-5 C_{18}) and at 22 mL/min (VP 250/21 nucleosil 300-7 C_{18}) with UV monitoring at 214 nm and 250 nm by using a linear A–B gradient (buffer A: 0.09% CF_3CO_2H in water; buffer B: 0.09% CF_3CO_2H in 90% acetonitrile). Each glycopeptide was analyzed by mass spectrometry by using electrospray ionization on an Esquire 3000+ Bruker Daltonics in positive mode. ^{1}H NMR and G-COSY were recorded in D_2O at 400 MHz with a Bruker Avance 400 spectrometer.

1. *Analytical data for compound* **5-Man**

Crude RP-HPLC profile (5 to 100% B in 20 min, λ = 250 nm)

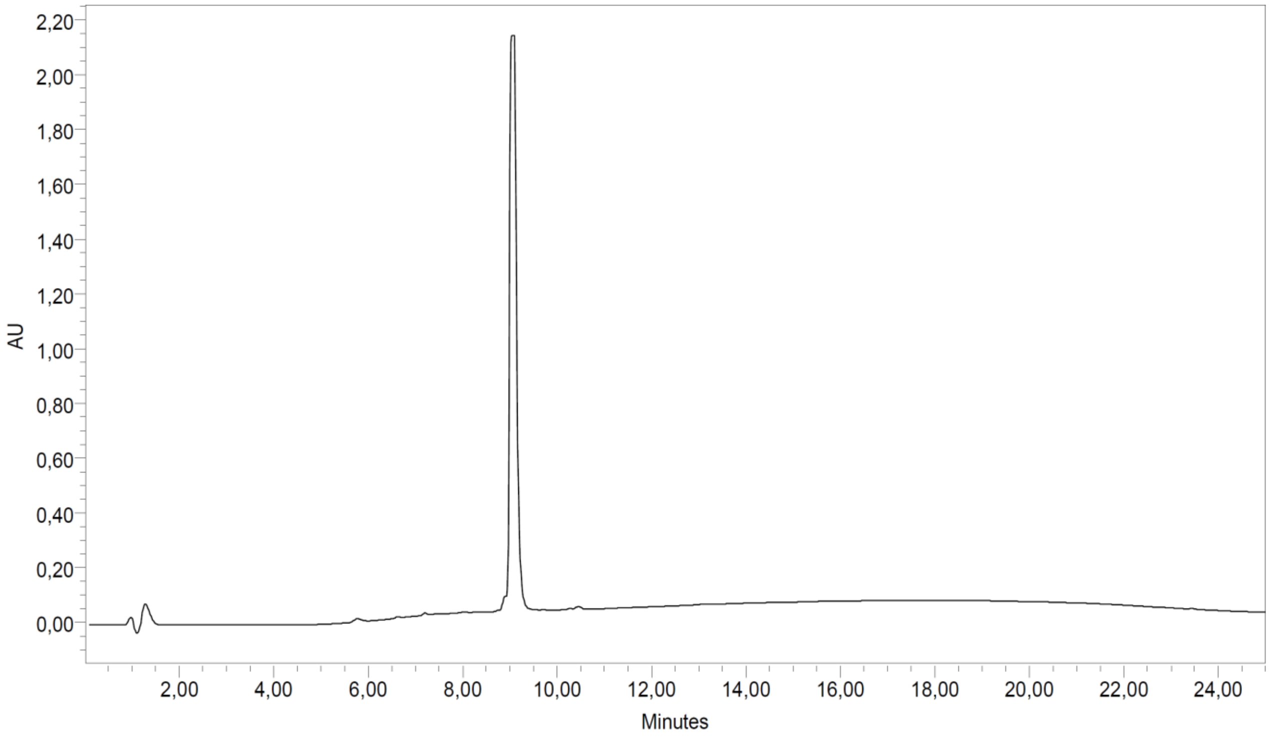

ESI-MS (positive mode): calcd for $C_{60}H_{97}N_{20}O_{24}$ 1481.7, found: *m/z* 1481.9 $[M + H]^+$

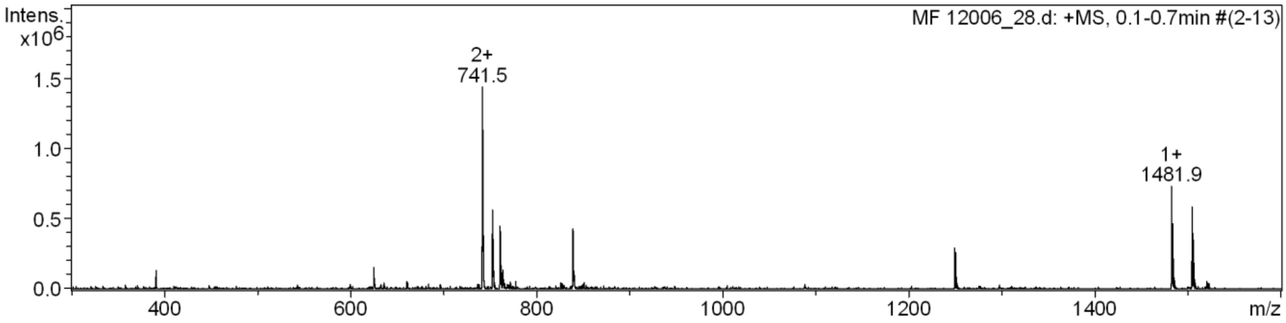

2. Analytical data for compound **5-Fuc**

Crude RP-HPLC profile (5 to 100% B in 20 min, λ = 250 nm)

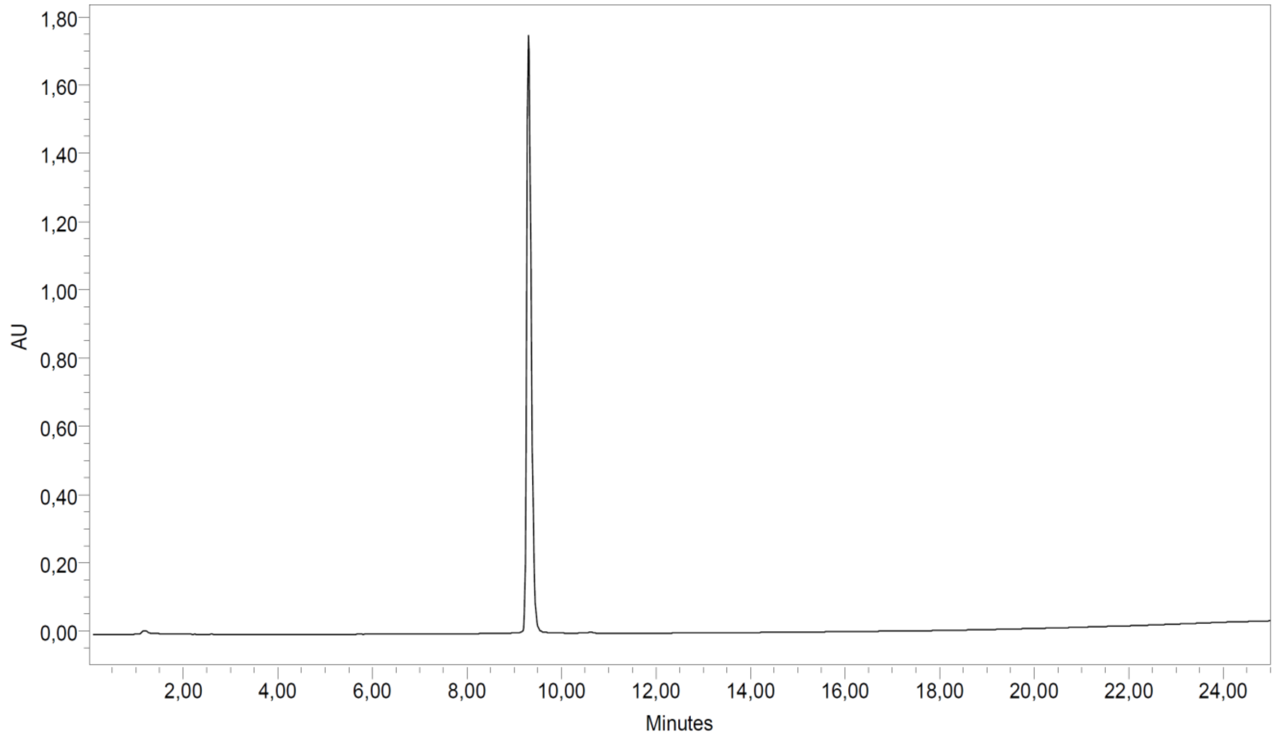

ESI-MS (positive mode): calcd for $C_{60}H_{96}N_{20}O_{22}Na$ 1471.7, found: *m*/*z* 1471.8 $[M + Na]^+$

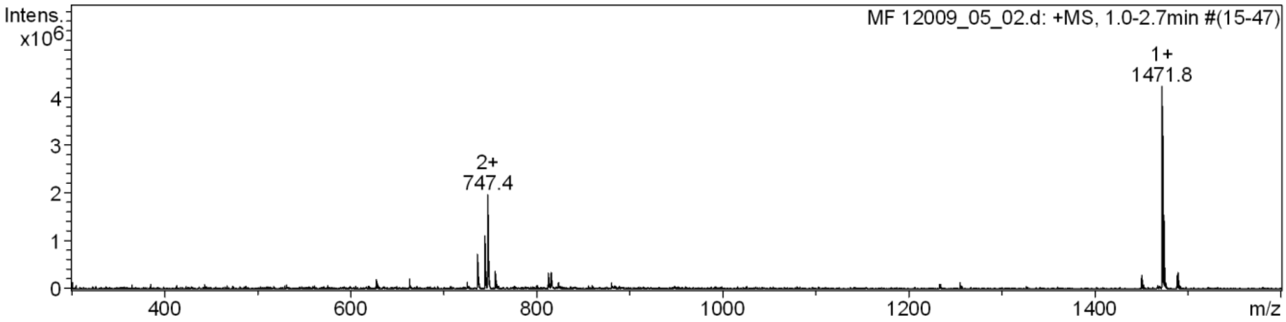

3. *Analytical data for compound* **5-Lac**

Crude RP-HPLC profile (5 to 100% B in 20 min, λ = 250 nm)

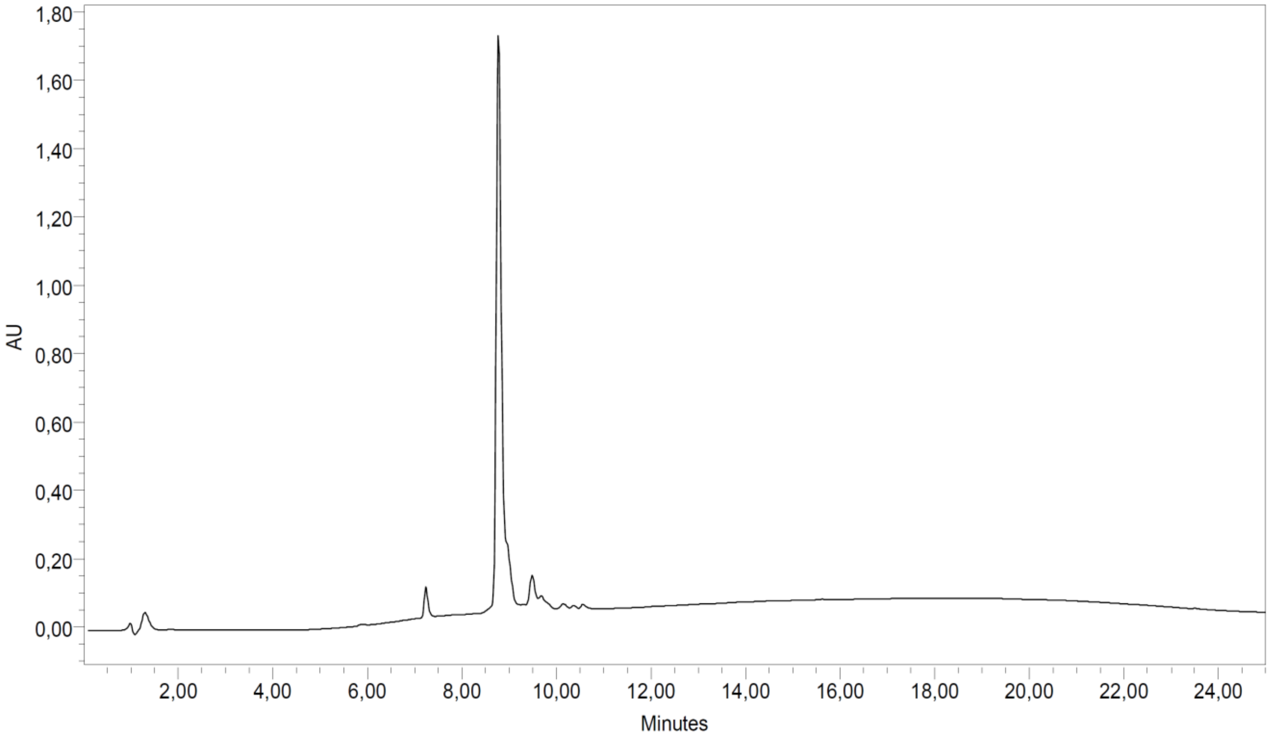

ESI-MS (positive mode): calcd for $C_{72}H_{117}N_{20}O_{34}$ 1805.8, found: *m*/*z* 1806.0 $[M + H]^+$

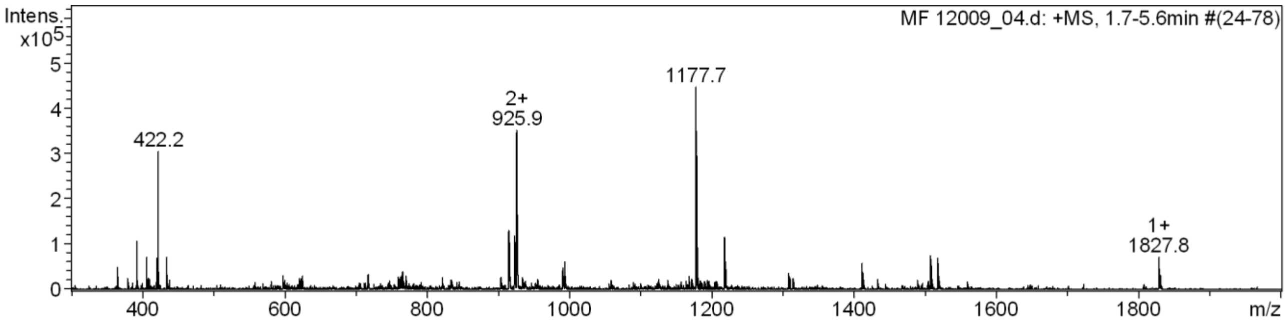

4. *Analytical data for compound* **6-Man/Fuc**

Crude RP-HPLC profile (5 to 100% B in 20 min, λ = 250 nm)

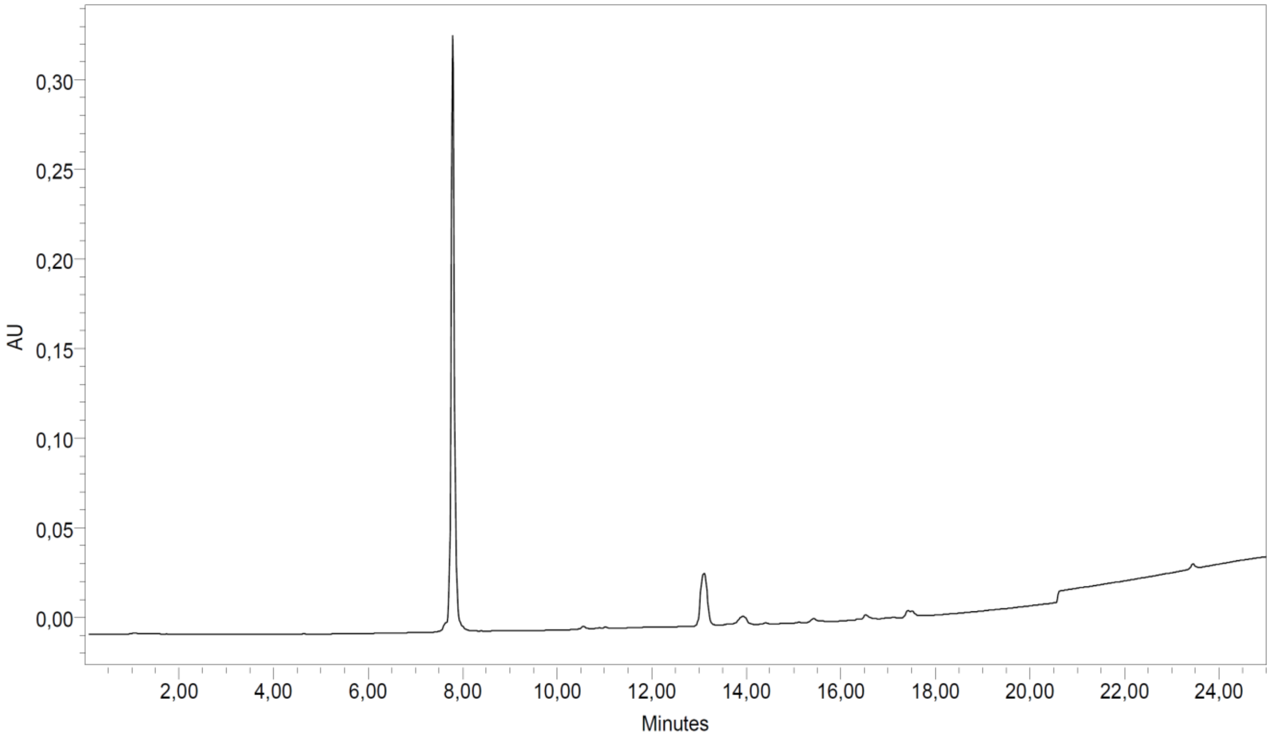

ESI-MS (positive mode): calcd for $C_{78}H_{124}N_{20}O_{34}$ 1885.9, found: *m/z* 1886.0[M + H]$^+$

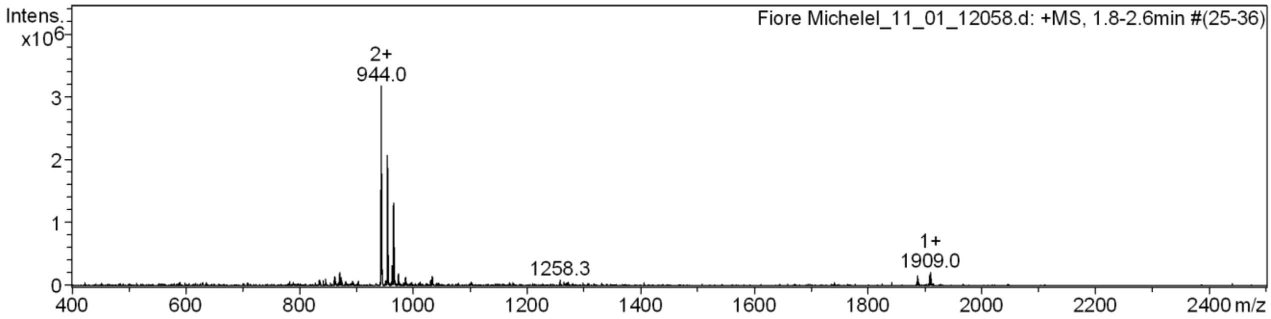

5. *Analytical data for compound* **6-Man/Lac**

Crude RP-HPLC profile (5 to 100% B in 20 min, λ = 250 nm)

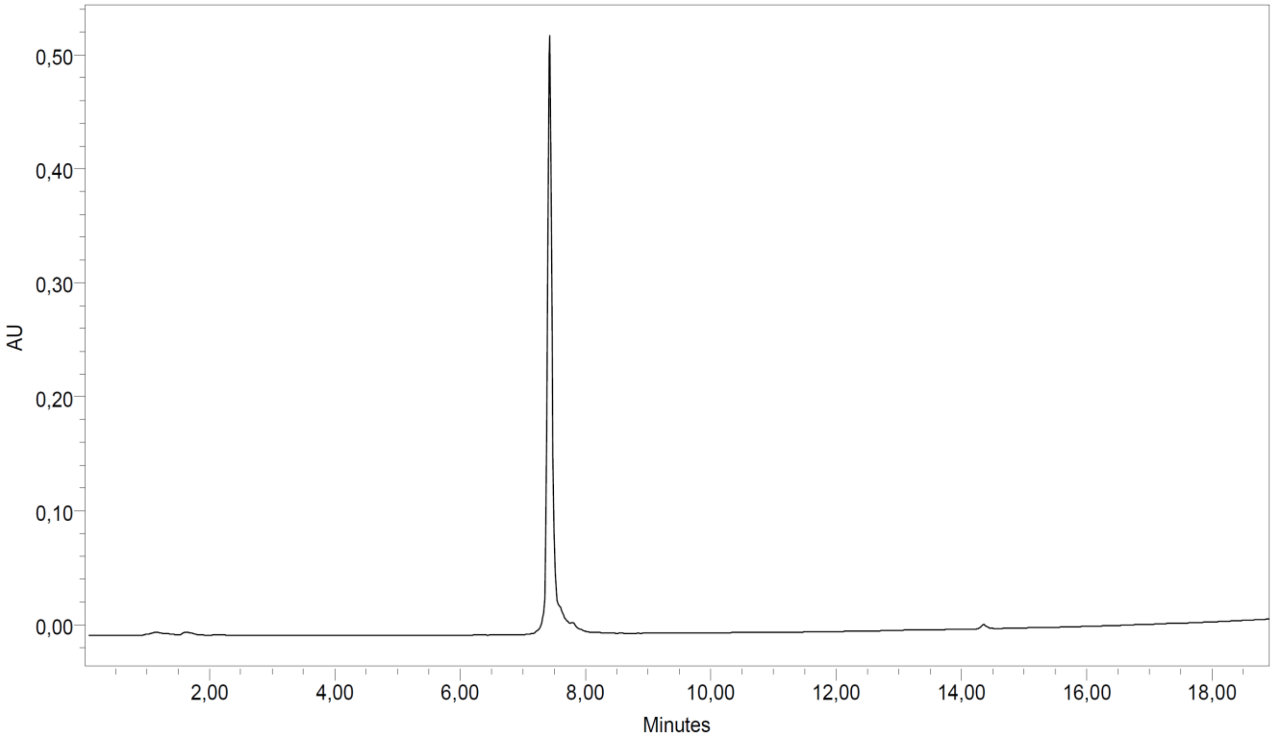

ESI-MS (positive mode): calcd for $C_{90}H_{144}N_{20}O_{46}$ 2242.9, found: *m/z* 2242.3 $[M + H]^+$

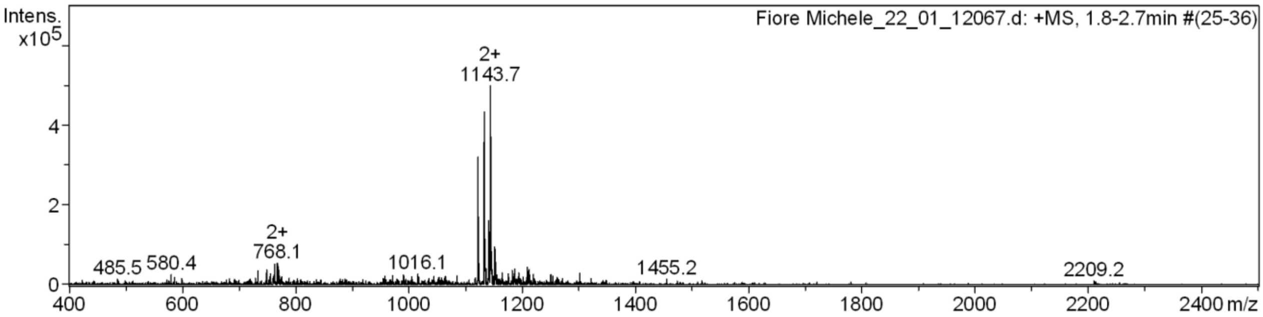

6. *Analytical data for compound* **6-Fuc/Man**

Crude RP-HPLC profile (5 to 100% B in 20 min, λ = 250 nm)

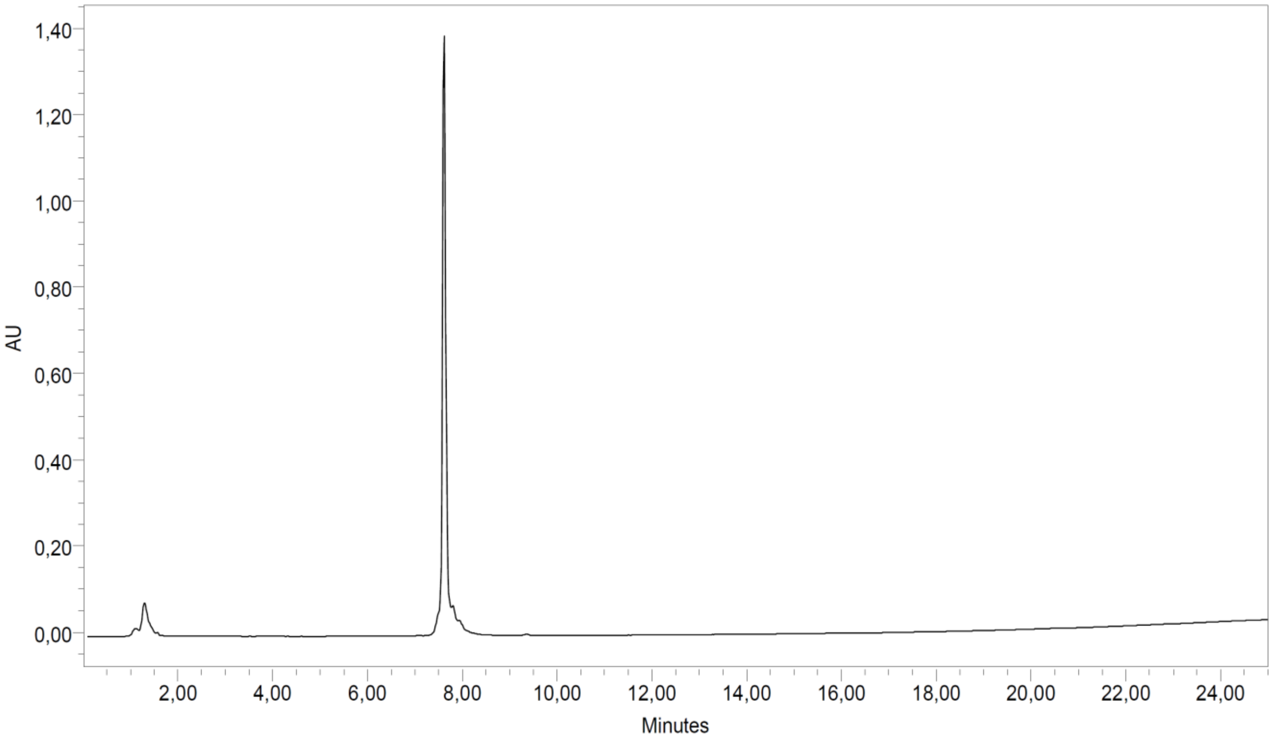

ESI-MS (positive mode): calcd for $C_{78}H_{124}N_{20}O_{34}$ 1885.9, found: *m*/*z* 1886.0 $[M + H]^+$

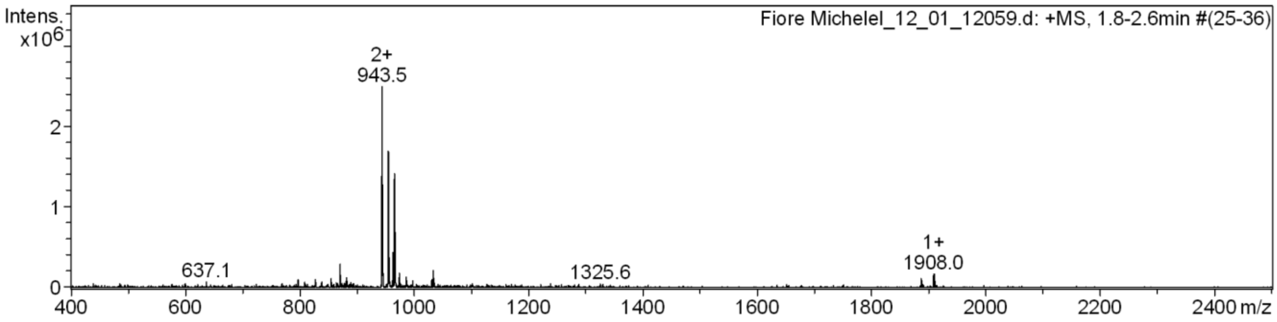

^1H NMR (D_2O, 400 MHz)

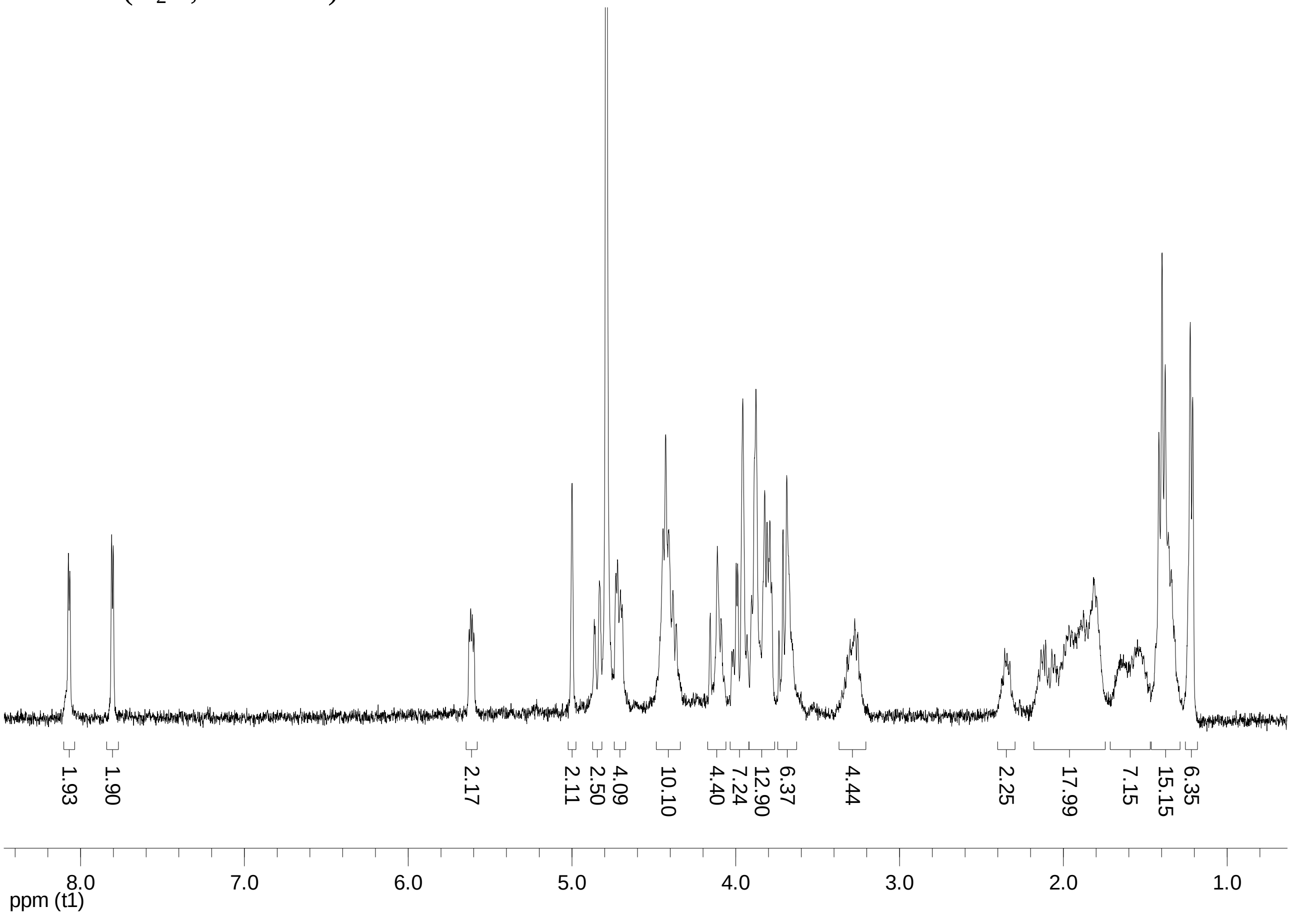

G-COSY (D_2O, 400 MHz)

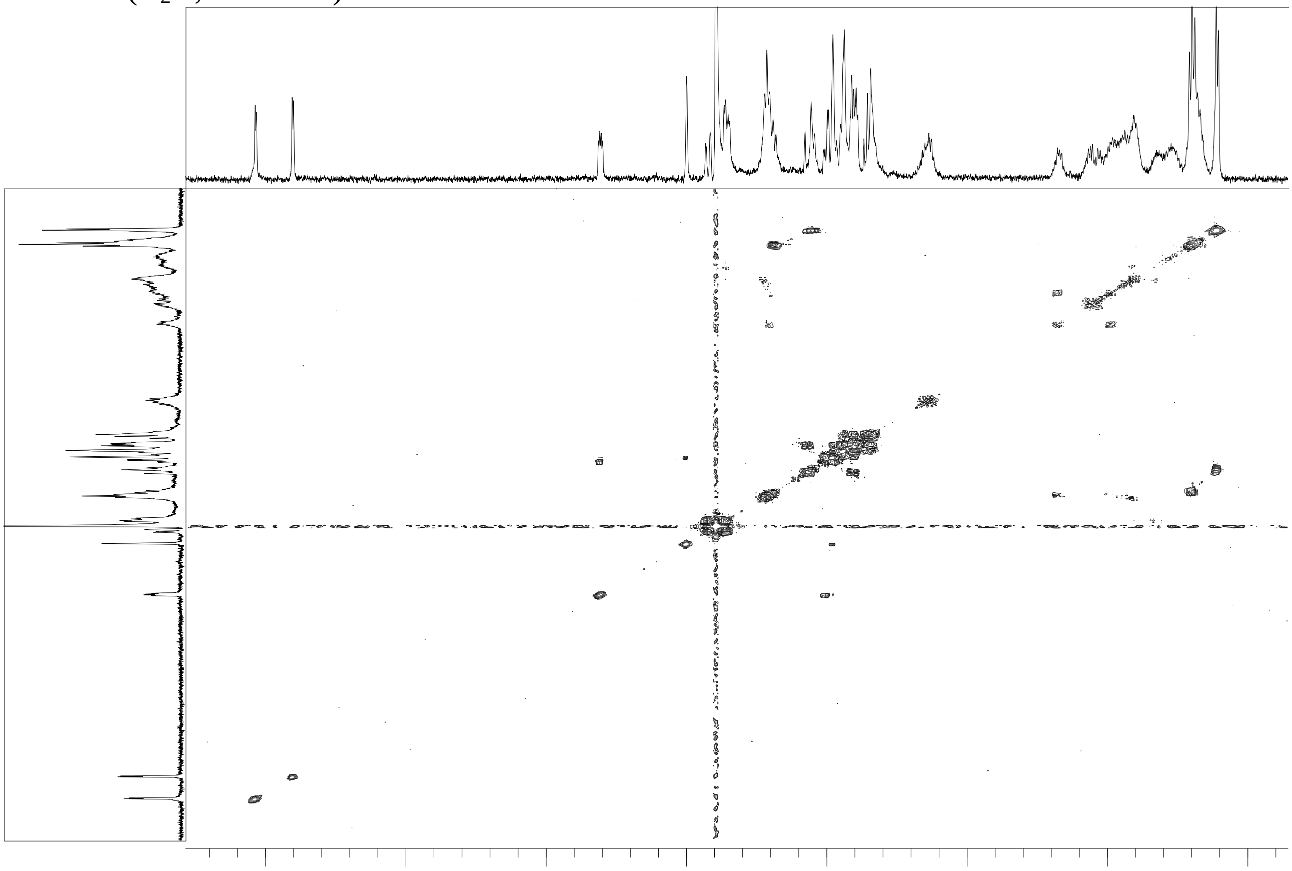

7. Analytical data for compound **6-Fuc/Lac**

Crude RP-HPLC profile (5 to 100% B in 20 min, λ = 250 nm)

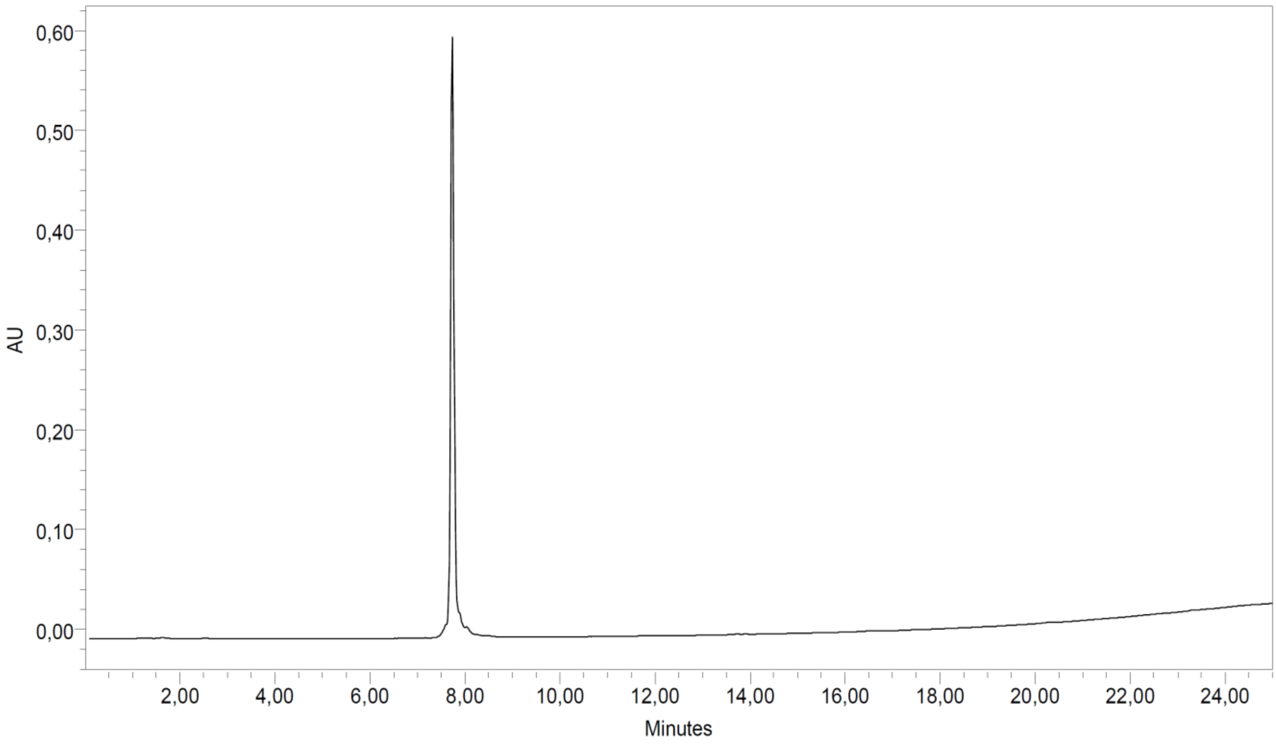

ESI-MS (positive mode): calcd for $C_{90}H_{144}N_{20}O_{44}$ 2210.0, found: *m/z* 2210.3 $[M + H]^+$

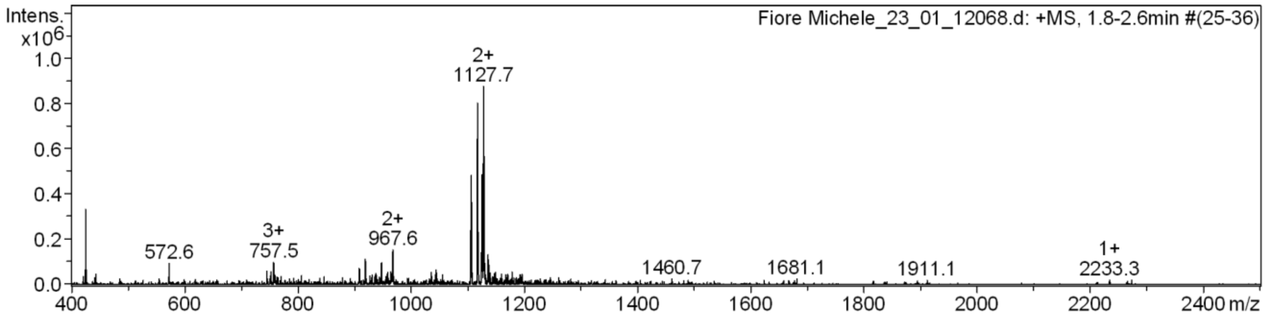

8. *Analytical data for compound* **6-Lac/Man**

Crude RP-HPLC profile (5 to 100% B in 20 min, λ = 250 nm)

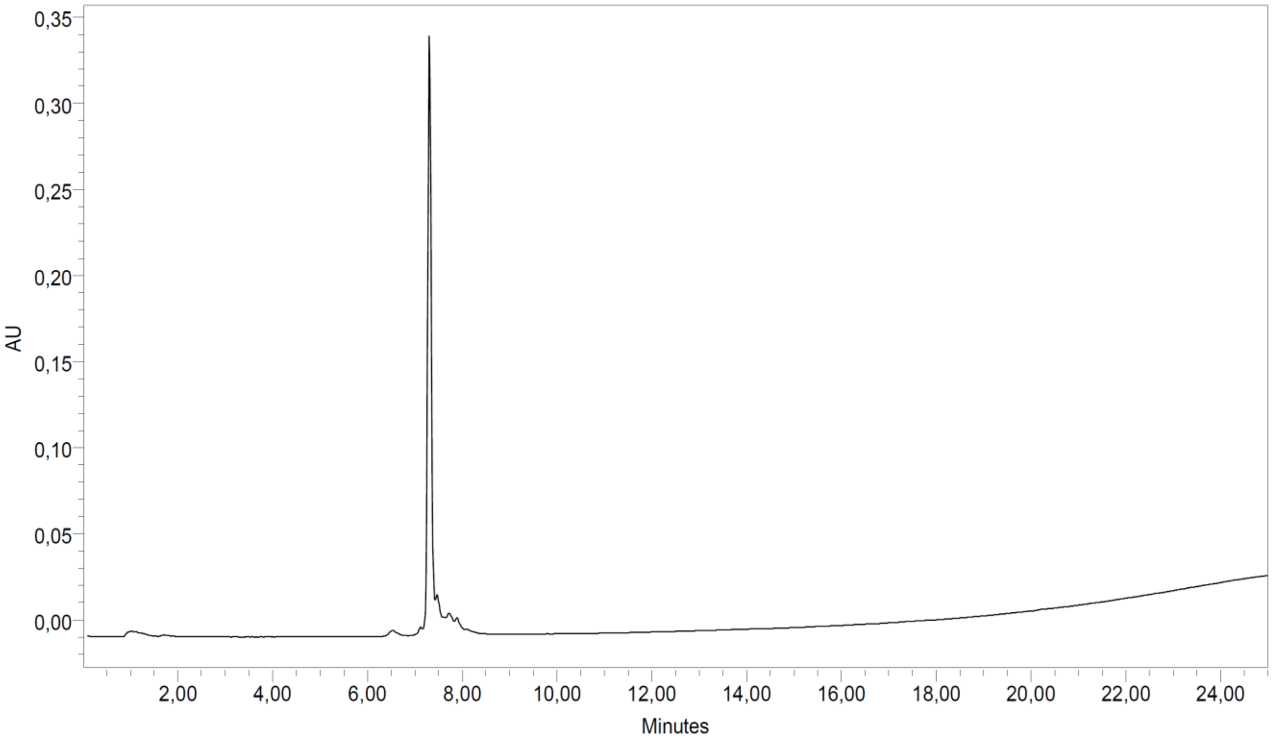

ESI-MS (positive mode): calcd for $C_{90}H_{145}N_{20}O_{46}$ 2242.9, found: *m*/*z* 2242.2 $[M + H]^+$

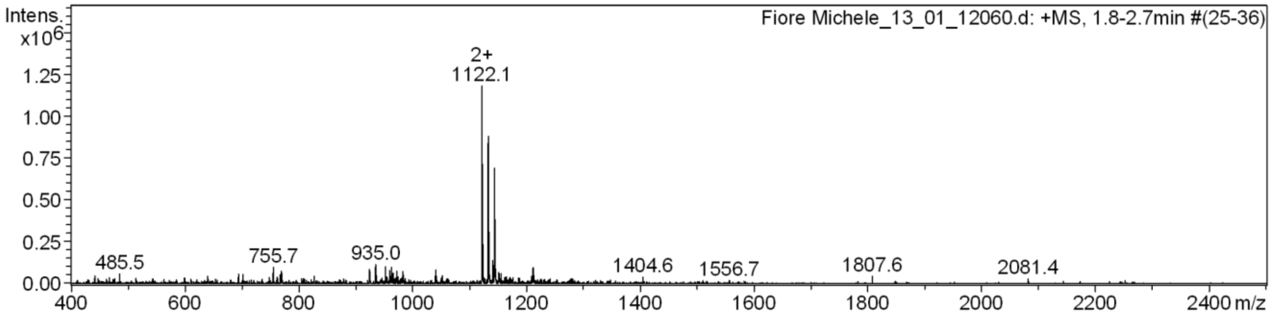

9. *Analytical data for compound* **6-Lac/Fuc**

Crude RP-HPLC profile (5 to 100% B in 20 min, λ = 250 nm)

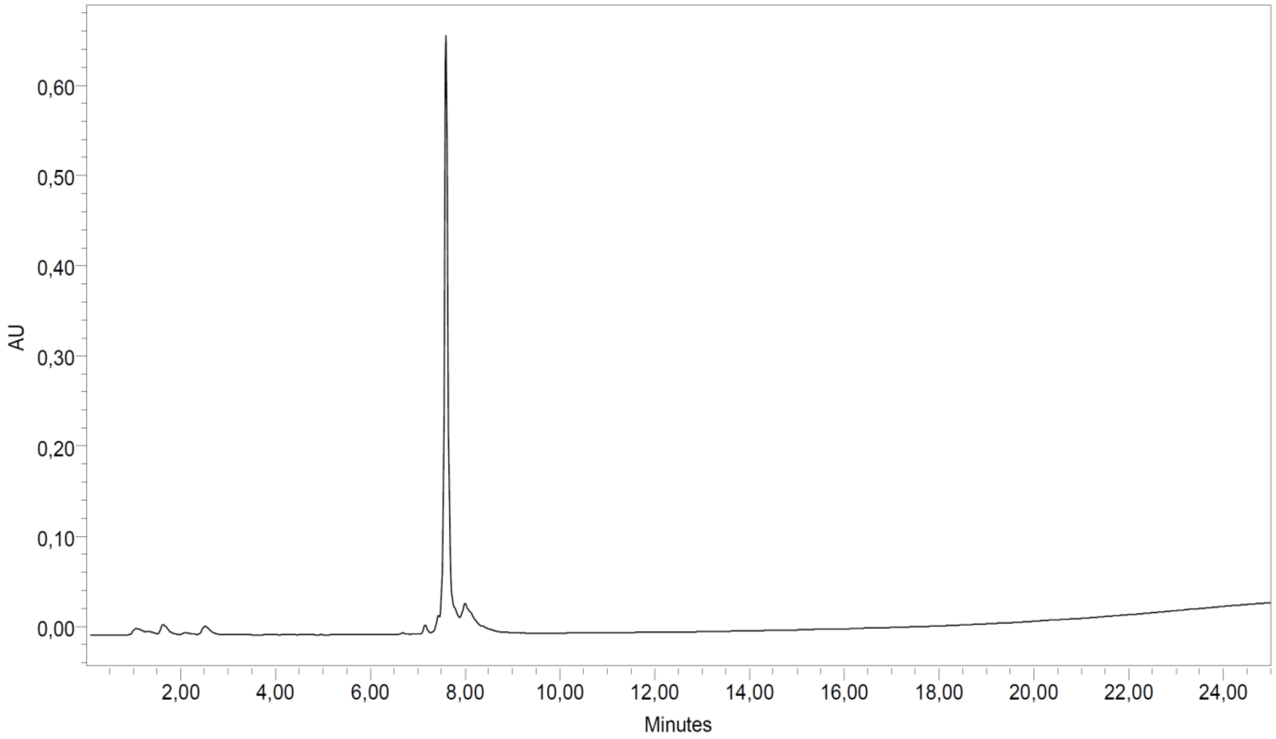

ESI-MS (positive mode): calcd for $C_{90}H_{145}N_{20}O_{44}$ 2210.0, found: *m*/*z* 2210.3 $[M + H]^+$

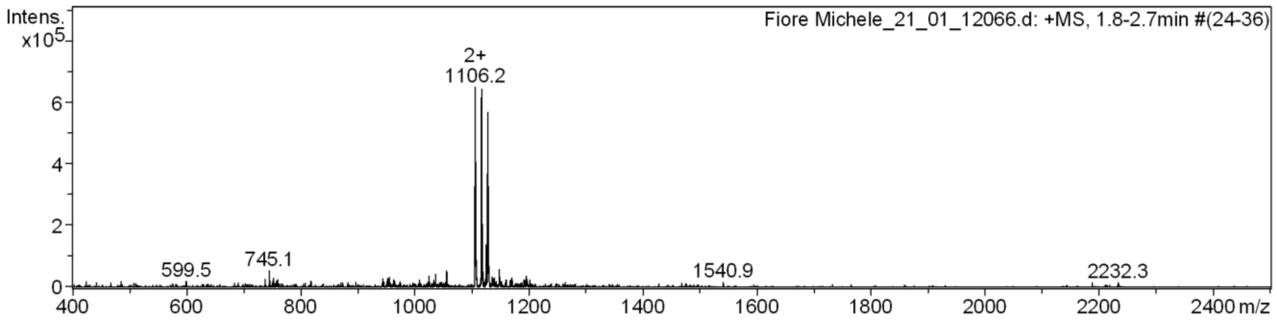

10. *Analytical data for compound* **7-Man**

Crude RP-HPLC profile (5 to 100% B in 20 min, λ = 250 nm)

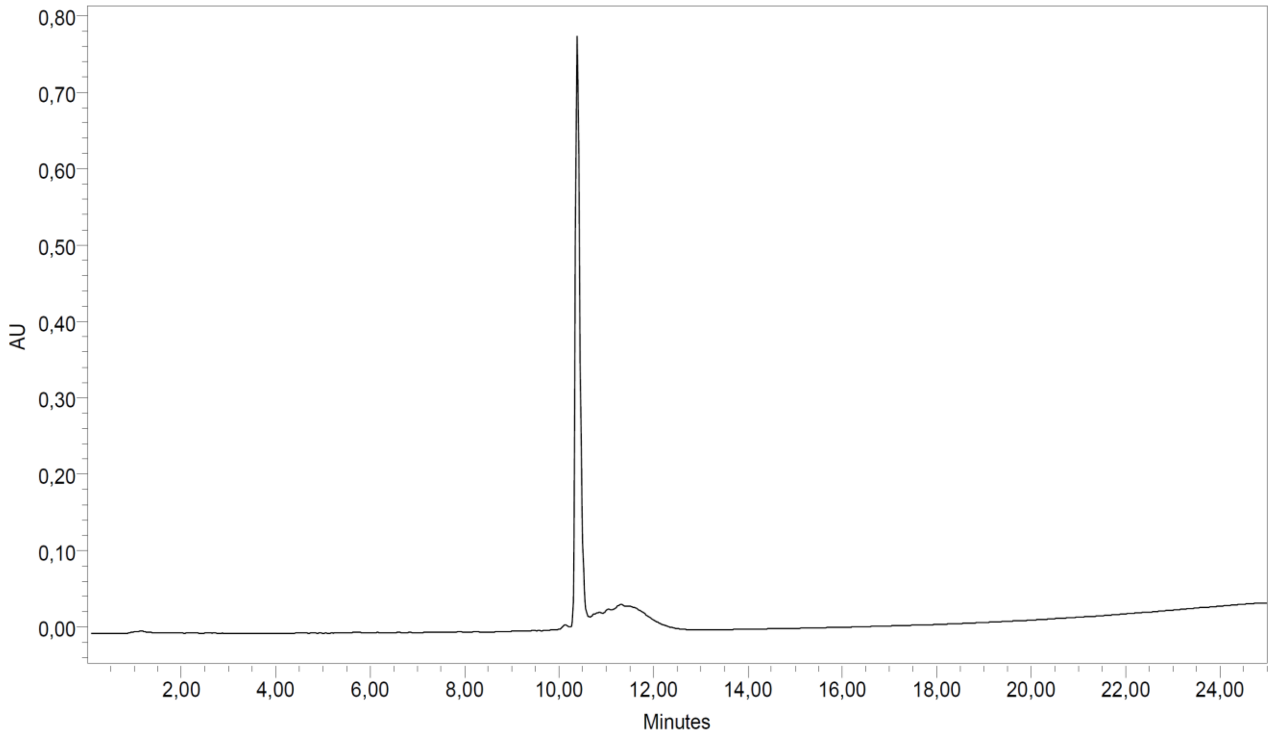

ESI-MS (positive mode): calcd for $C_{52}H_{84}N_{21}O_{16}$ 1258.6, found: *m/z* 1258.8 $[M + H]^+$

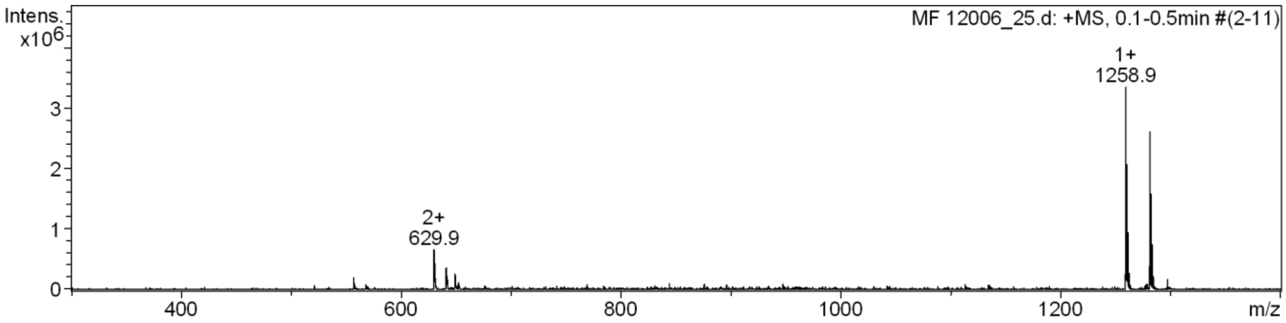

11. Analytical data for compound **7-Fuc**

Crude RP-HPLC profile (5 to 100% B in 20 min, λ = 250 nm)

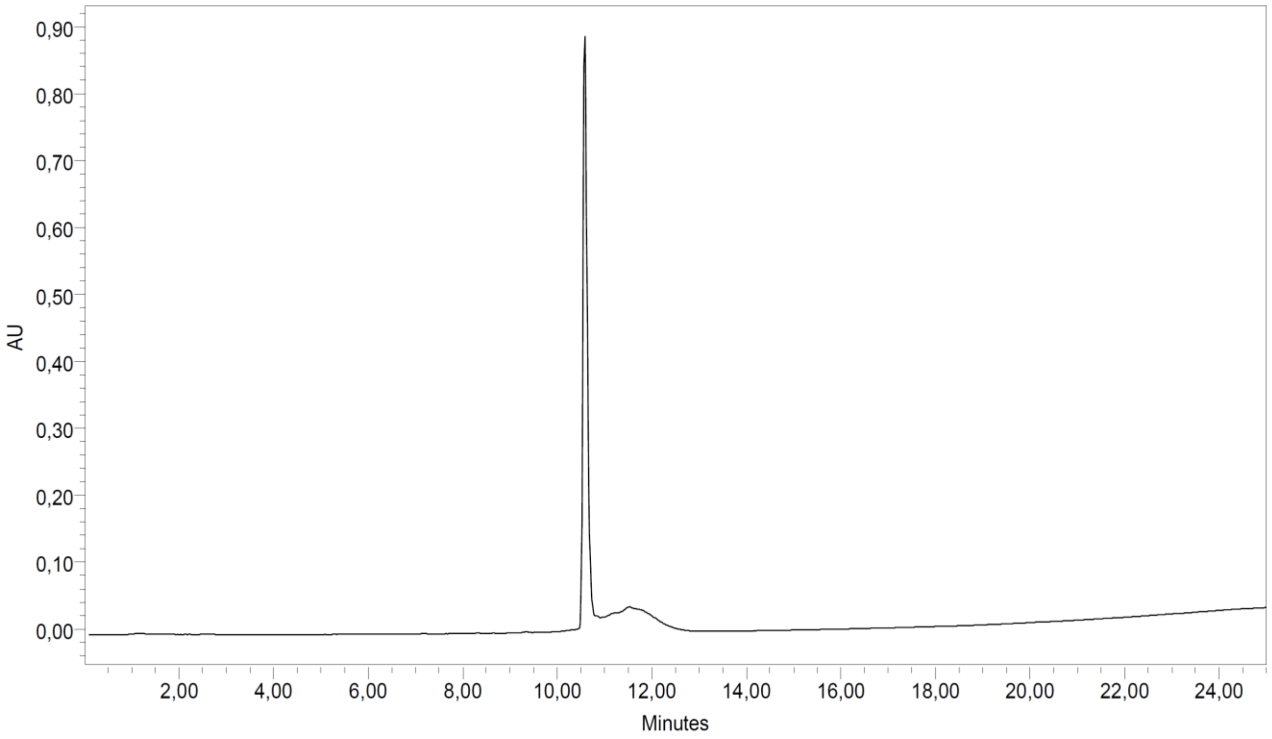

ESI-MS (positive mode): calcd for $C_{52}H_{84}N_{21}O_{17}$ 1274.6, found: *m/z* 1274.8 $[M + H]^+$

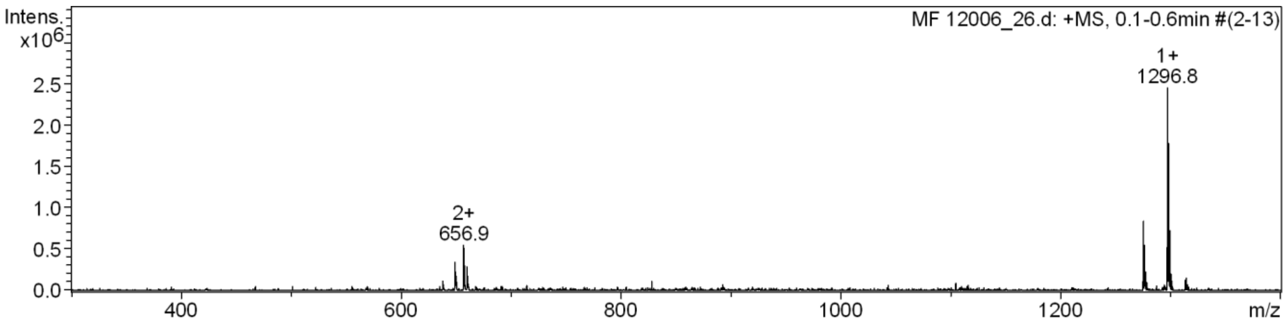

12. Analytical data for compound **7-Lac**

Crude RP-HPLC profile (5 to 100% B in 20 min, λ = 250 nm)

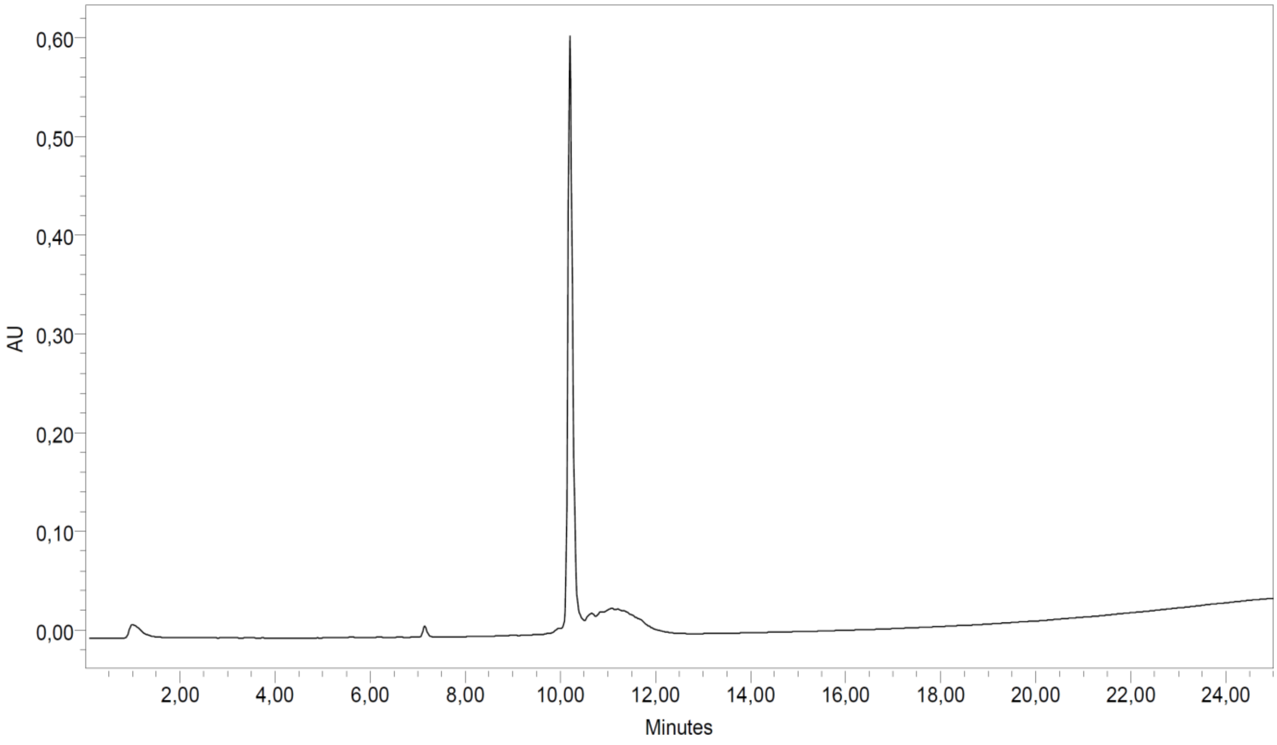

ESI-MS (positive mode): calcd for $C_{58}H_{94}N_{21}O_{22}$ 1436.7, found: *m/z* 1436.9 $[M + H]^+$

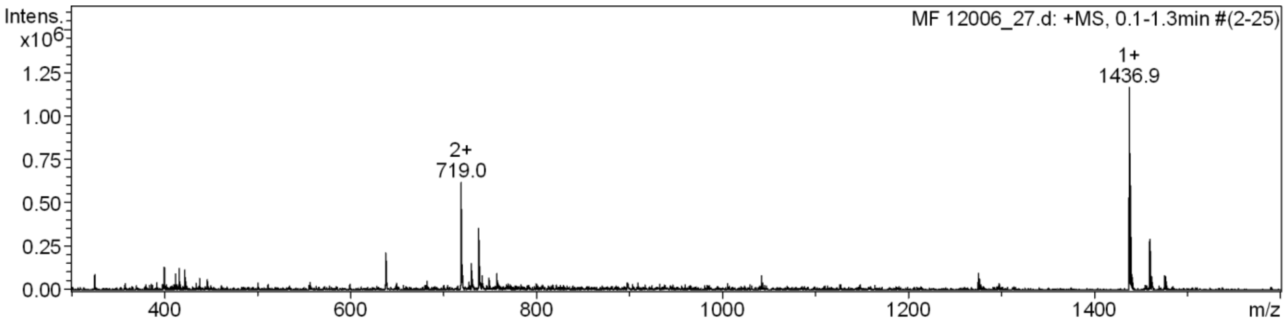

13. Analytical data for compound **8-Man/Fuc**

Crude RP-HPLC profile (5 to 100% B in 20 min, λ = 250 nm)

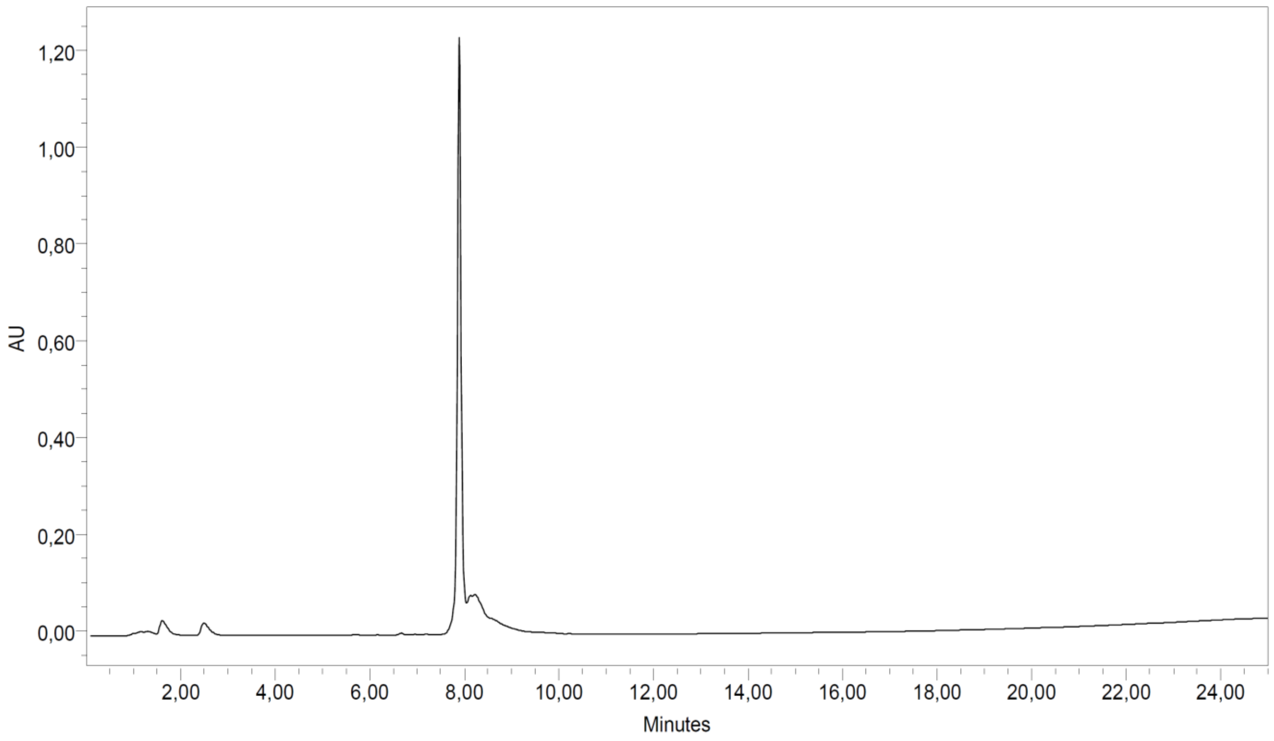

ESI-MS (positive mode): calcd for $C_{79}H_{126}N_{21}O_{32}$ 1880.9, found: *m*/*z* 1881.1 [M + H]$^+$

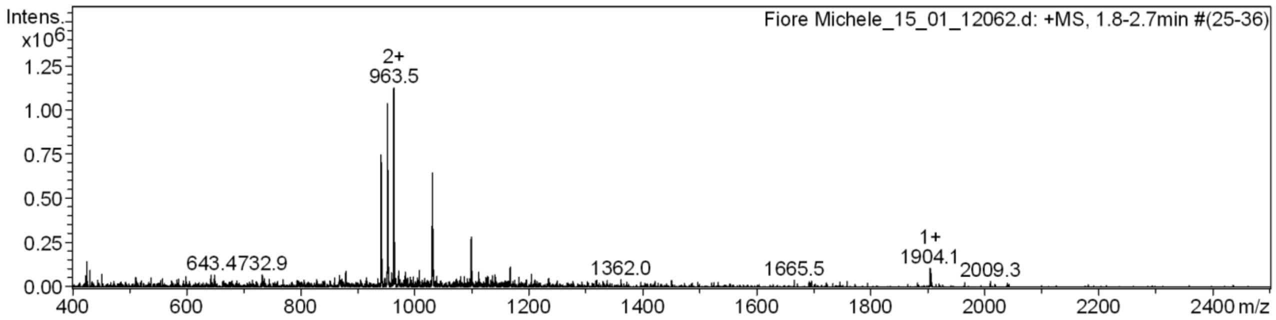

14. Analytical data for compound **8-Man/Lac**

Crude RP-HPLC profile (5 to 100% B in 20 min, λ = 250 nm)

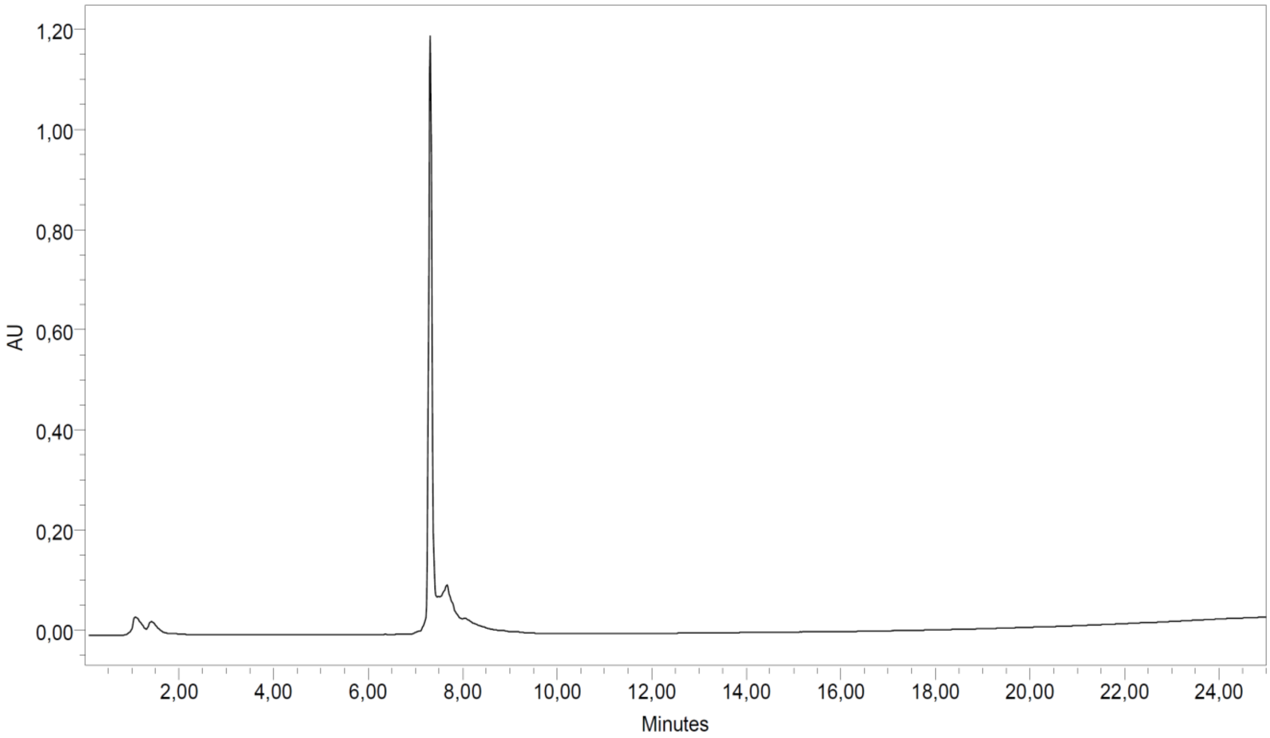

ESI-MS (positive mode): calcd for $C_{97}H_{156}N_{21}O_{50}$ 2415.0, found: *m/z* 2415.4 $[M + H]^+$

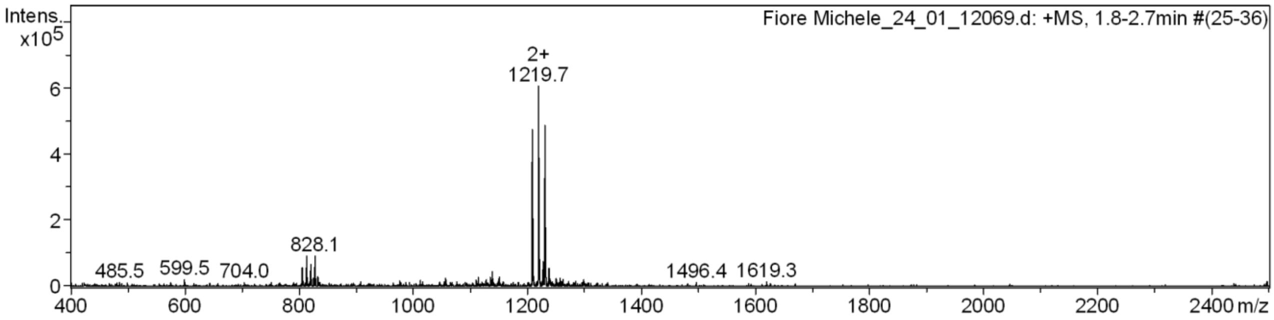

15. Analytical data for compound **8-Fuc/Man**

Crude RP-HPLC profile (5 to 100% B in 20 min, λ = 250 nm)

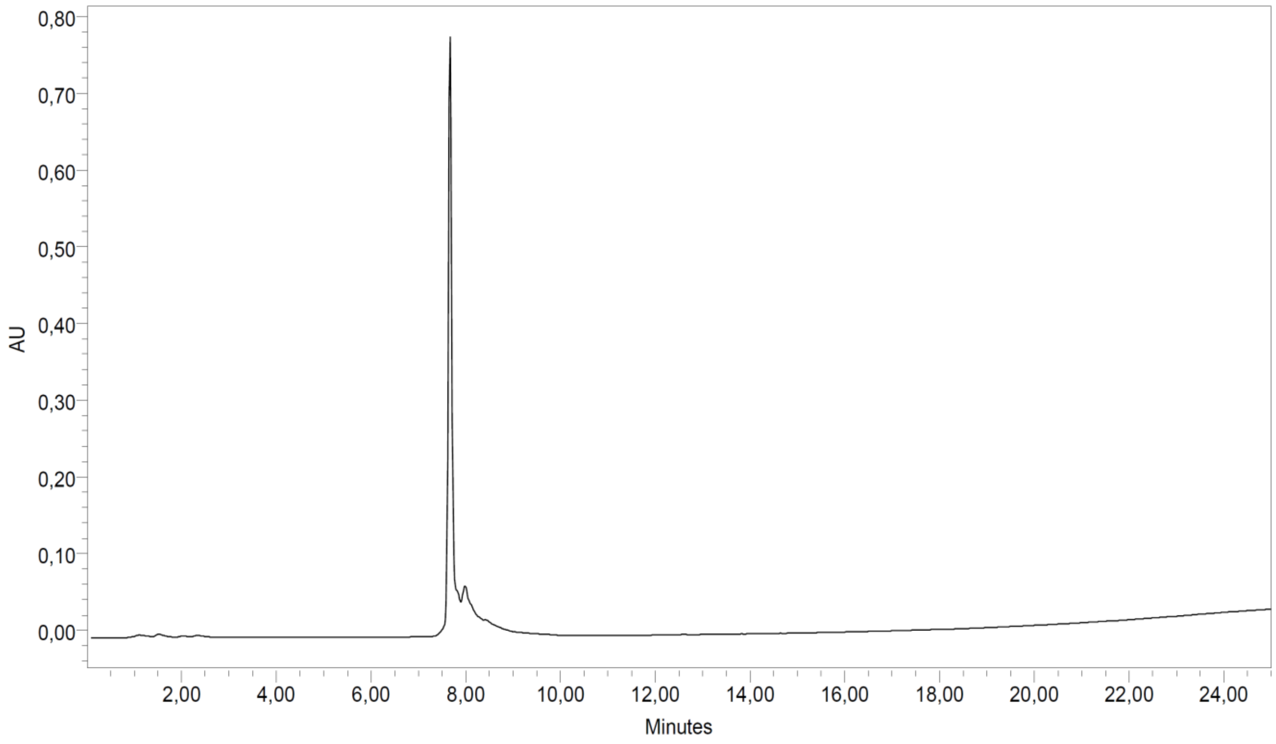

ESI-MS (positive mode): calcd for $C_{79}H_{126}N_{21}O_{34}$ 1912.9, found: *m/z* 1913.2 $[M + H]^+$

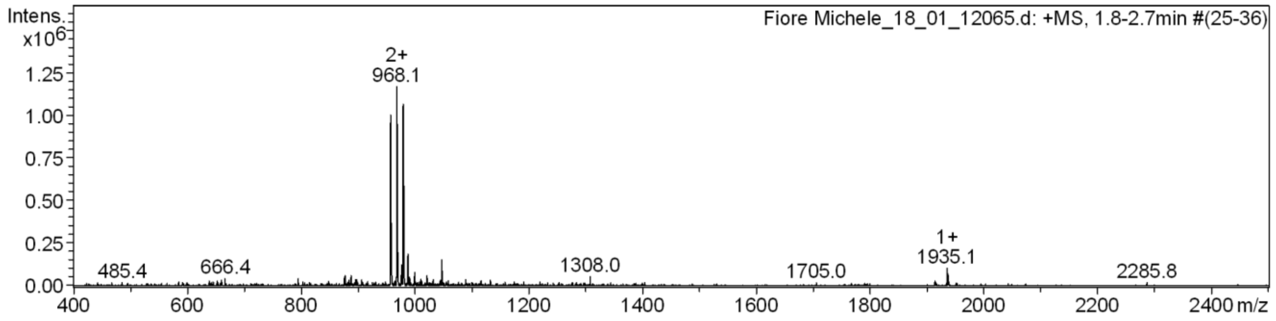

^{1}H NMR (D_2O, 400 MHz)

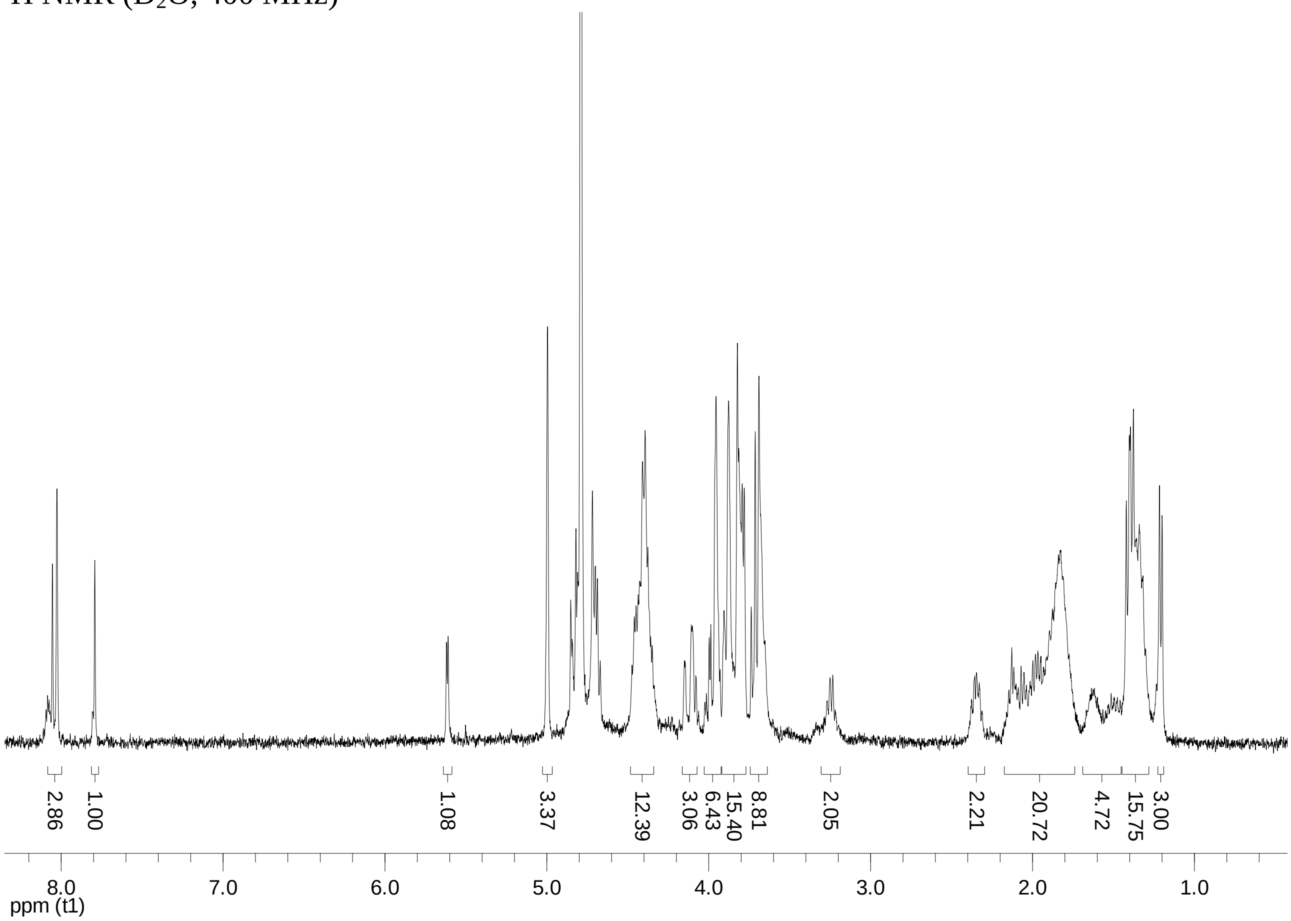

G-COSY (D_2O, 400 MHz)

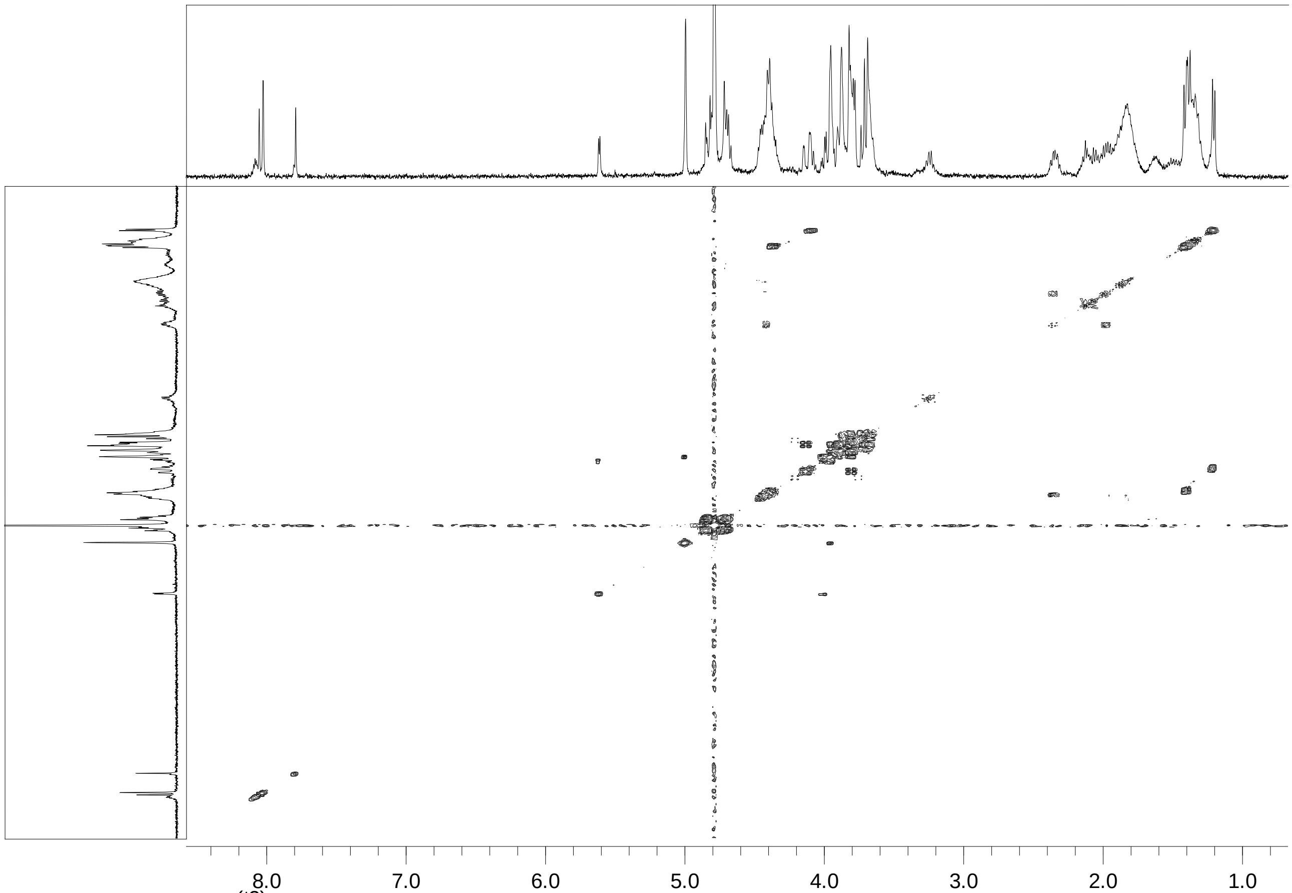

16. Analytical data for compound **8-Fuc/Lac**

Crude RP-HPLC profile (5 to 100% B in 20 min, λ = 250 nm)

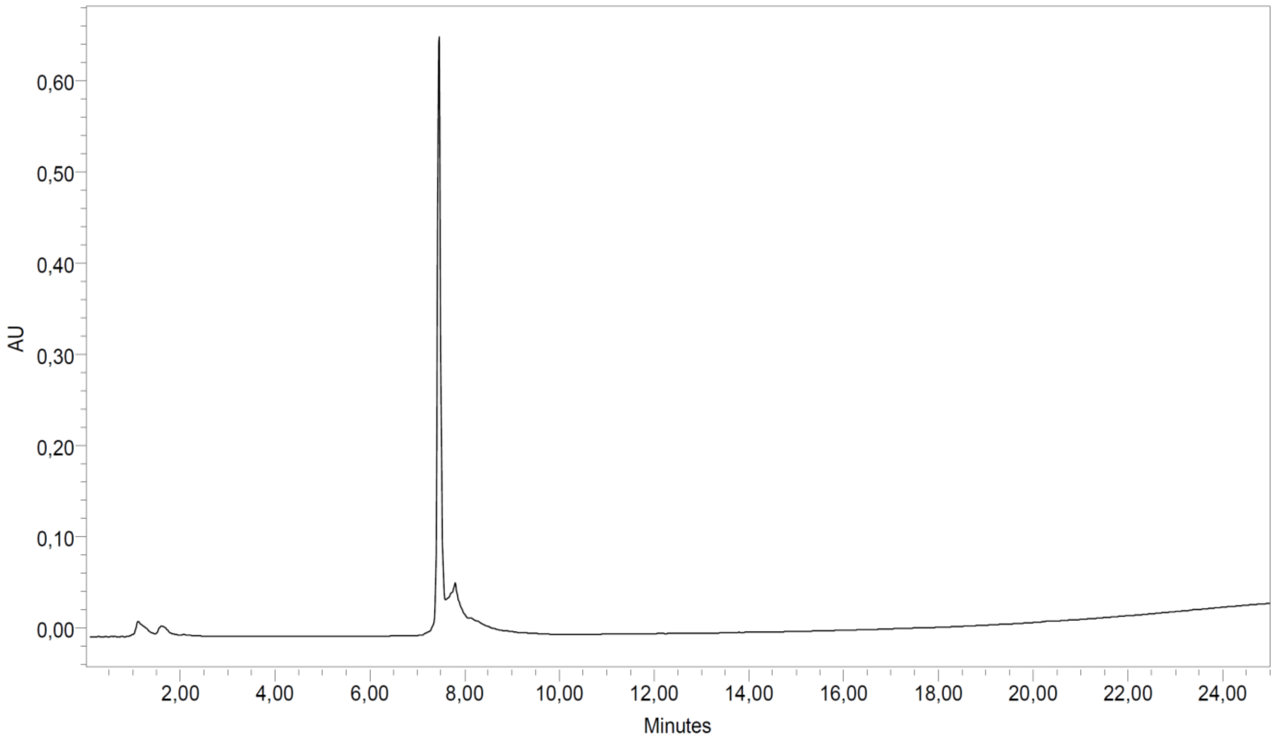

ESI-MS (positive mode): calcd for $C_{97}H_{156}N_{21}O_{49}$ 2400.0, found: *m/z* 2399.0 $[M + H]^+$

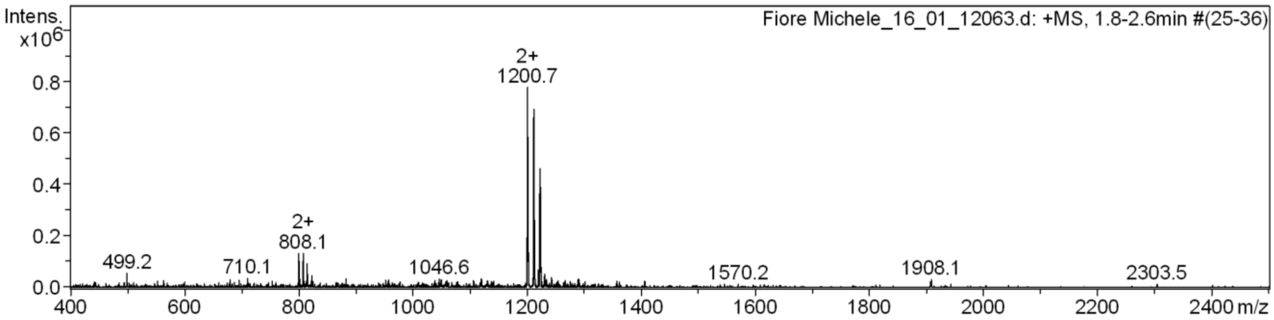

17. Analytical data for compound **8-Lac/Man**

Crude RP-HPLC profile (5 to 100% B in 20 min, λ = 250 nm)

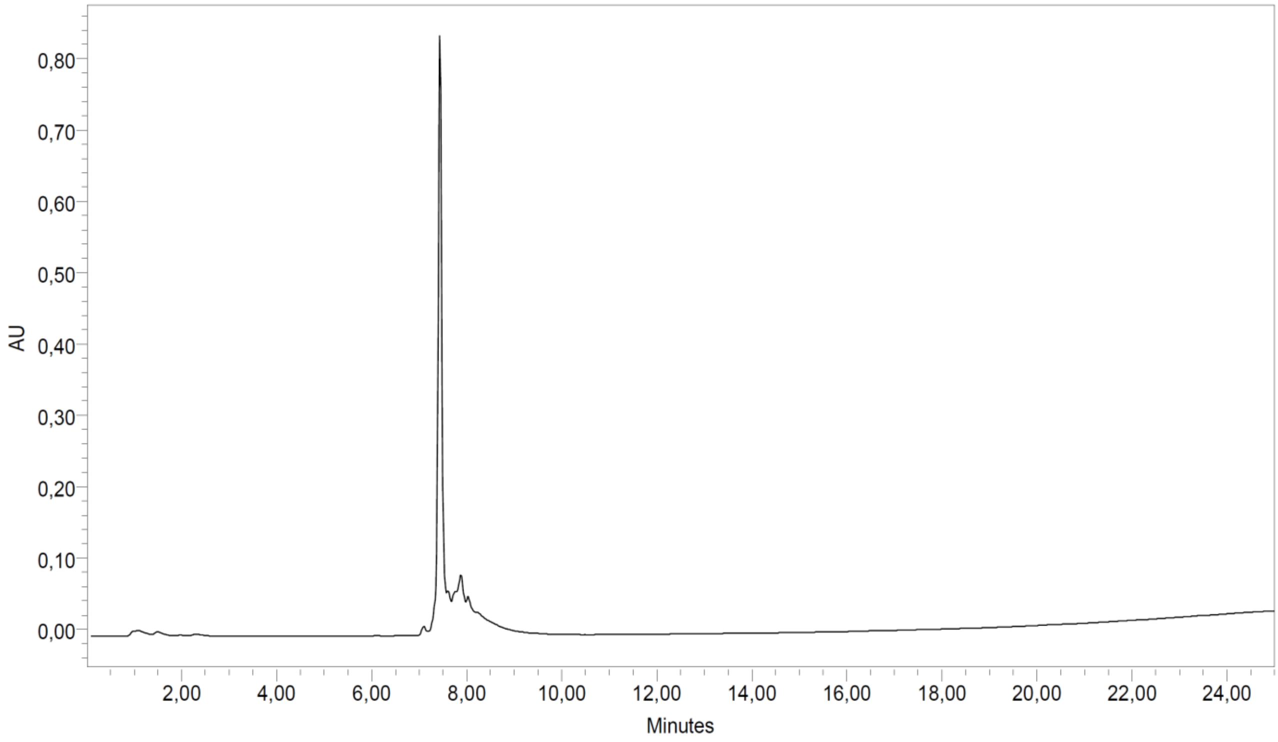

ESI-MS (positive mode): calcd for $C_{85}H_{136}N_{21}O_{40}$ 2092.0, found: *m/z* 2091.2 $[M + H]^+$

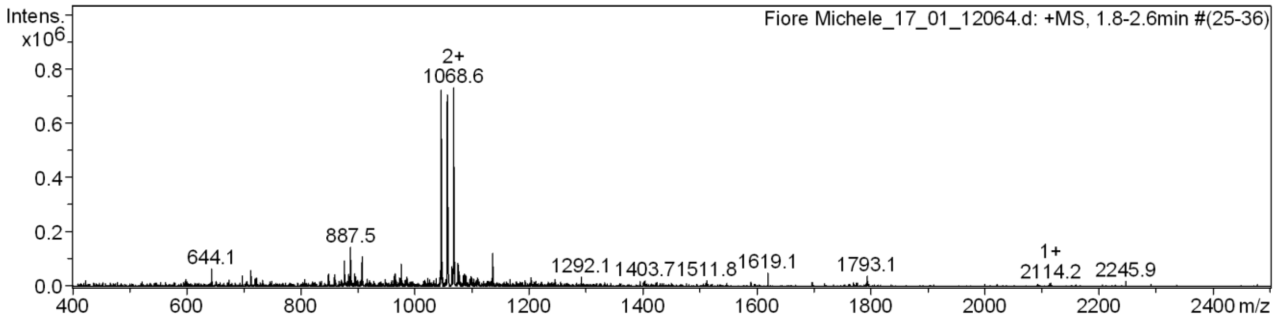

18. Analytical data for compound **8-Lac/Fuc**

Crude RP-HPLC profile (5 to 100% B in 20 min, λ = 250 nm)

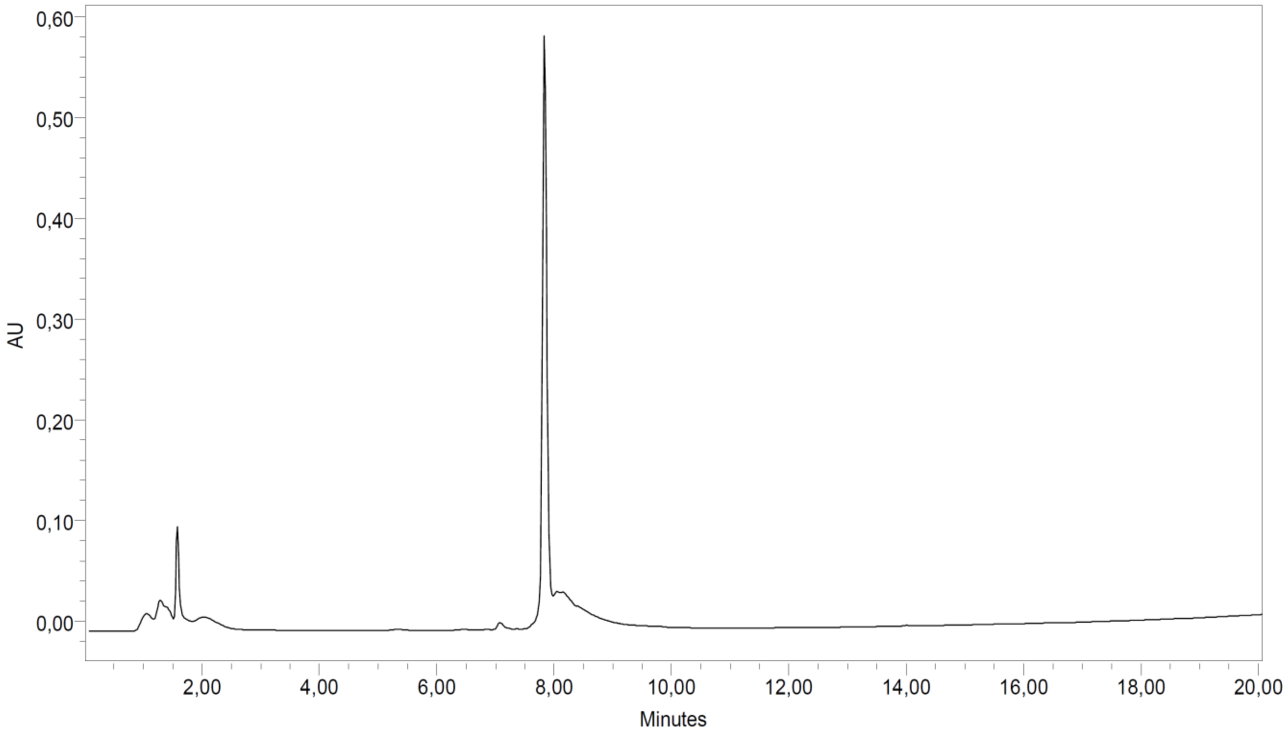

ESI-MS (positive mode): calcd for $C_{85}H_{136}N_{21}O_{37}$ 2043.1, found: *m/z* 2043.9 $[M + H]^+$

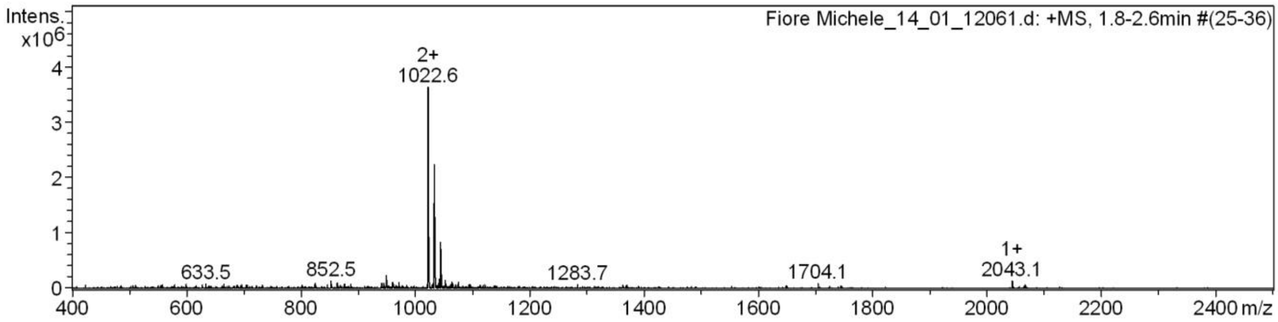

Supplement: File 1 — Crude RP-HPLC profiles and ESI-MS spectra for the heteroglycoclusters. [file Beilstein_J_Org_Chem-08-421-s001.pdf]
